# Genome-wide identification of microRNA targets reveals positive regulation of the Hippo pathway by miR-122 during liver development

**DOI:** 10.1038/s41419-021-04436-7

**Published:** 2021-12-14

**Authors:** Yin Zhang, Ye-Ya Tan, Pei-Pei Chen, Hui Xu, Shu-Juan Xie, Shi-Jun Xu, Bin Li, Jun-Hao Li, Shun Liu, Jian-Hua Yang, Hui Zhou, Liang-Hu Qu

**Affiliations:** 1 MOE Key Laboratory of Gene function and regulation, State Key Laboratory of Biocontrol, School of Life Sciences, Sun Yat-Sen University, Guangzhou, 510275 China; 2 Guangdong Provincial Key Laboratory of Malignant Tumor Epigenetics and Gene Regulation, Research Center of Medicine, Sun Yat-sen Memorial Hospital, Sun Yat-sen University, Guangzhou, 510120 China; 3 Guangdong Province Hospital of Chinese Medicine, AMI Key Laboratory of Chinese Medicine in Guangzhou, , The Second Affiliated Hospital of Guangzhou University of Chinese Medicine, Guangdong Provincial Academy of Chinese Medical Science, Guangzhou, 510006 China

**Keywords:** Differentiation, miRNAs

## Abstract

Liver development is a highly complex process that is regulated by the orchestrated interplay of epigenetic regulators, transcription factors, and microRNAs (miRNAs). Owing to the lack of global in vivo targets of all miRNAs during liver development, the mechanisms underlying the dynamic control of hepatocyte differentiation by miRNAs remain elusive. Here, using Argonaute (Ago) high-throughput sequencing of RNA isolated by crosslinking immunoprecipitation (HITS-CLIP) in the mouse liver at different developmental stages, we characterized massive Ago-binding RNAs and obtained a genome-wide map of liver miRNA-mRNA interactions. The dynamic changes of five clusters of miRNAs and their potential targets were identified to be differentially involved at specific stages, a dozen of high abundant miRNAs and their epigenetic regulation by super-enhancer were found during liver development. Remarkably, miR-122, a liver-specific and most abundant miRNA in newborn and adult livers, was found by its targetome and pathway reporter analyses to regulate the Hippo pathway, which is crucial for liver size control and homeostasis. Mechanistically, we further demonstrated that miR-122 negatively regulates the outcomes of the Hippo pathway transcription factor TEAD by directly targeting a number of hippo pathway regulators, including the coactivator TAZ and a key factor of the phosphatase complex PPP1CC, which contributes to the dephosphorylation of YAP, another coactivator downstream of the Hippo pathway. This study identifies for the first time the genome-wide miRNA targetomes during mouse liver development and demonstrates a novel mechanism of terminal differentiation of hepatocytes regulated by the miR-122/Hippo pathway in a coordinated manner. As the Hippo pathway plays important roles in cell proliferation and liver pathological processes like inflammation, fibrosis, and hepatocellular carcinoma (HCC), our study could also provide a new insight into the function of miR-122 in liver pathology.

## Introduction

The liver is a highly organized structure formed by the interaction of many cells and tissues. Liver development is orchestrated by numerous transcription factors, epigenetic regulators, and microRNAs (miRNAs). MiRNAs are key regulators in cell signaling pathways and play important roles in cell differentiation and organ development [1–4]. An increasing number of studies have revealed that liver cell differentiation is accompanied by the strictly dynamic regulation of numerous miRNAs [5]. More importantly, each miRNA may regulate multiple targets involved in different signaling pathways in a spatial and temporal manner. To date, several hundred miRNAs with different abundances have been identified in liver cells; however, owing to the lack of global in vivo targets of all these miRNAs during liver development, the mechanisms underlying the dynamic control of the differentiation of hepatocytes and liver morphogenesis by miRNAs remain elusive.

The Hippo pathway is a critical regulator of liver organ size, homeostasis, and pathological states such as HCC [6–9]. The activity of the Hippo pathway is continually elevated for the maintenance of differentiated hepatocyte state during the liver development, while inactivation of Hippo pathway in vivo is sufficient to dedifferentiate adult hepatocytes into progenitor-like cells [10, 11]. In the canonical Hippo pathway, Yap is phosphorylated by the protein kinase cascade, leading to the exit of Yap from the nucleus, which inhibits its coactivator function. Phosphatase dephosphorylates YAP, which results in its nuclear accumulation and promotes cell proliferation through working together with TEAD transcription factors [7]. The outcome of the Hippo pathway can also be regulated by the other molecules that influence the transcriptional activities of TEAD [7]. The coordinated dynamic change in Hippo signaling and miRNAs, as well as the regulation of the pathway activity by miRNAs during liver development, is a very interesting question to explore. Moreover, previous studies have showed that miR-122 is not only the major regulator of terminal differentiation during the liver development, but also an important tumor suppressor [12–14]. Knockout of miR-122 resulted in a high incidence of HCC in mouse [15, 16]. However, the underlying mechanisms, especially the details of key pathways regulated by miR-122 were not fully elucidated. Deeply and systematically analysis of miR-122 targets may provide clues to discover new mechanisms for controlling liver tumorigenesis.

Ago HITS-CLIP and its variant technologies provide good opportunities for the systematic study of miRNA targets in vivo [17]. In this study, we conducted Ago HITS-CLIP and performed genome-wide identification of miRNA targets of the mouse liver at different developmental stages. We found the coordination of epigenetic regulation signals with the dynamic changes of high abundant miRNAs during liver development, and we also characterized five clusters of miRNAs and their targetomes respectively. Importantly, miR-122 was identified as a key regulator for the terminal differentiation of hepatocytes via regulating the Hippo pathway. This study provides comprehensive microRNA targetomes during mouse liver development and demonstrates a novel mechanism of terminal differentiation of hepatocytes regulated by the miR-122- Hippo pathway in a coordinated manner.

## Results

### Characterization of Ago-binding RNAs in the developing livers of mice

**Fig. 1:**
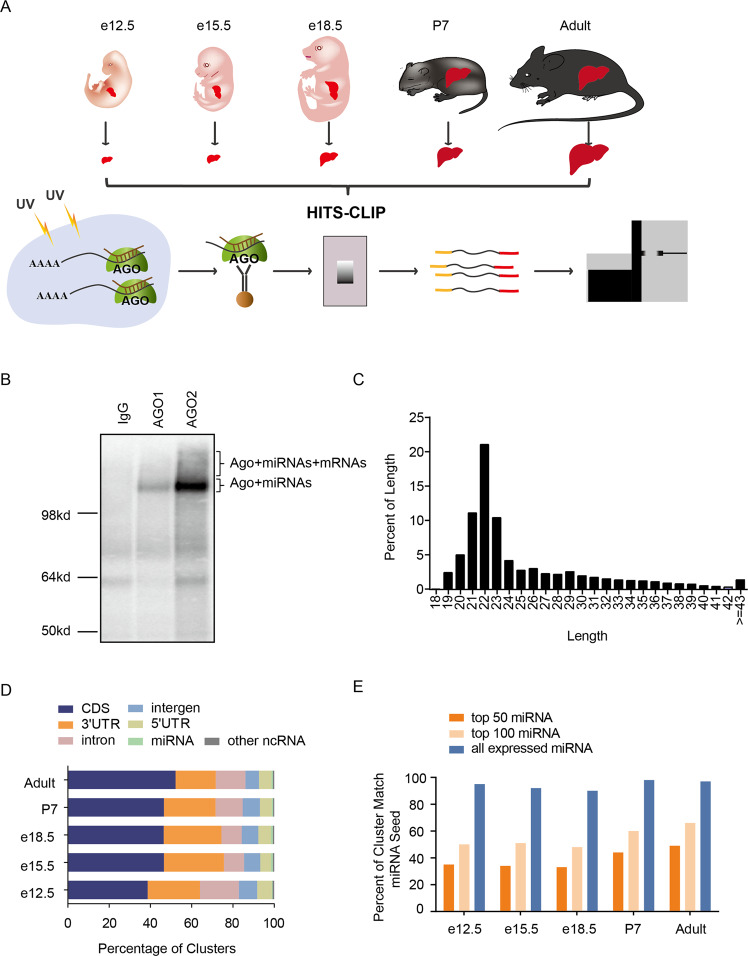
Genome-wide identification and characterization of miRNA-target interactions in developing mouse livers by Ago HITS-CLIP. **A** Workflow for performing Ago HITS-CLIP of different stages of liver development. **B** Autoradiography results of Ago and Ago-binding RNAs generated by HITS-CLIP. **C** Length distribution of RNA tags from Ago HITS-CLIP. **D** Genome location distribution of Ago-binding RNA clusters. **E** Proportion of 3’UTR tags possessing miRNA seed sequences.

To explore the dynamic changes of Ago-binding miRNAs and their mRNA targets, the Ago HITS-CLIP technique was performed on mouse livers harvested from different developmental stages (Fig. 1A). After confirming the specific immunoprecipitation of Ago2 and Ago1 (Fig. S1), the Ago-binding RNAs were analyzed by autoradiography. One band representing Ago-bound miRNAs and an upper band representing Ago-bound mRNAs were observed (Figs. 1B, S2). Using high-throughput sequencing, more than 20 million reads were produced from each sample, with >70% mapped to the mouse genome (Table S1). The length distribution showed a peak at 22 nt, representing miRNAs (Fig. 1C), which was consistent with the autoradiography results.

We identified ~30,000 high-confidence mRNA clusters, ~40%, 30%, and 6% of which were mapped to CDSs, 3′UTRs, and 5′UTRs of mRNA, respectively (Figs. 1D, S3 and Table S2). Although many reports suggest that miRNAs function by preferentially binding at the 3′UTR of mRNAs, comparable levels of Ago bound to the CDS and 3′UTR of mRNAs have been observed in Ago CLIP experiments [18–21].

We next analyzed the relationship between Ago-binding mRNA clusters and miRNAs “seed region” present in the liver samples. We found that ~50% of the clusters in the 3′UTR, contained at least one seed-matched site to the top 100 expressed miRNAs and >90% of the clusters had targeting sites if all expressed miRNAs were considered (Fig. 1E).

Collectively, for the first time, we obtained comprehensive datasets with a total of more than one million of miRNA-mRNA interactions of mouse livers at five different developmental stages.

### Dynamic changes of high abundant miRNAs and their epigenetic regulation during mouse liver development

**Fig. 2:**
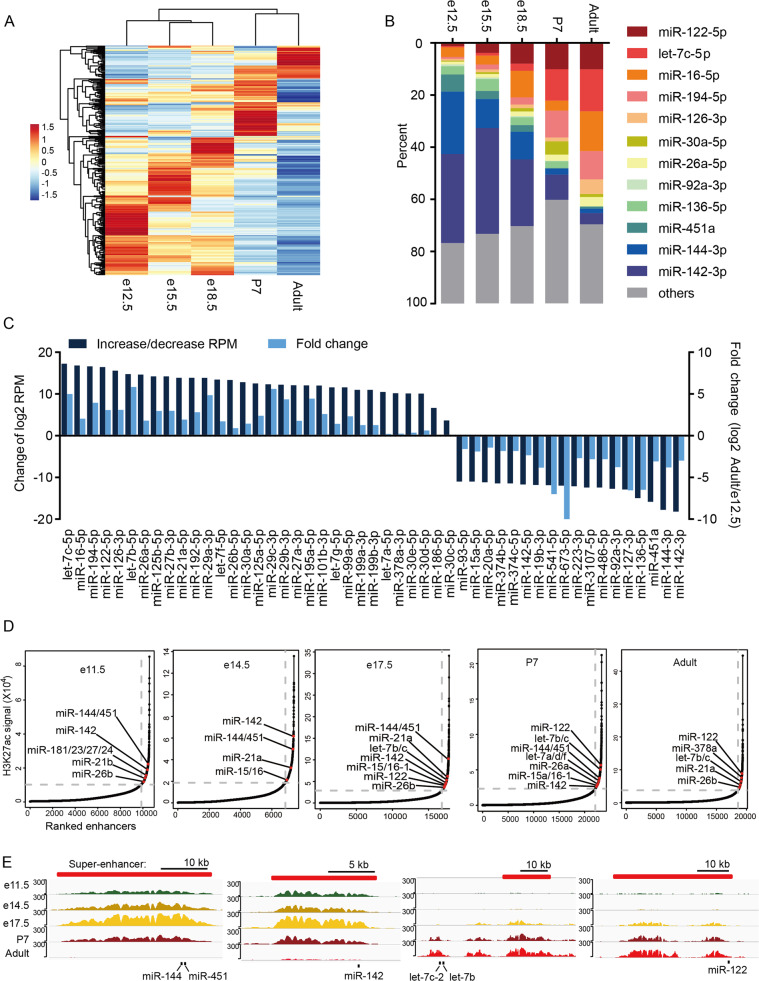
Dynamic changes of high abundant miRNAs and their epigenetic regulation during mouse liver development. **A** Cluster analysis of differentially expressed miRNAs during mouse liver development. **B** Changes in the most highly expressed miRNAs during mouse liver development. **C** Increase or decrease of miRNA of adult liver compared with e12.5; left *Y*-axis: increased or decreased FPKM (log2), right *Y*-axis: log2-fold change. **D** Hockey stick plots of H3K27ac signals of mouse liver at different developmental stages, super-enhancer-associated miRNAs are highlighted. **E** ChIP-seq profiles of H3K27ac near super-enhancer-associated miRNA loci of mouse liver at different developmental stages. The predicted super-enhancers regions are depicted as red bars. The *y*-axis represents reads per million (rpm) of ChIP-seq.

To characterize the expression of all miRNAs during liver development, hierarchical clustering analysis was used to reveal the dynamic changes of miRNA profiles (Fig. 2A). Based on the expression pattern of each miRNA, five miRNA clusters, with expression enriched in e12.5 (Cluster I), e15.5 (Cluster II), e18.5 (Cluster III), P7 (Cluster IV), and adult liver (Cluster V) were identified (Table S3). This profile implies that several hundred miRNAs, regardless of their abundance, are differentially involved in the specific stages of liver development. A dozen of highly expressed miRNAs that accounted for 60% of the total Ago-binding miRNAs were found to dramatically decrease or increase during the development (Fig. 2B). The most abundant miRNAs in clusters I and II are known hematopoiesis and stem cell-related miRNAs, such as miR-142, miR-144, miR-451, miR-136, and miR-92, constituting >60% of total of the miRNAs at early stages. After evacuation of the hematopoietic system from the fetal liver at e18.5, the expression of these miRNAs dropped sharply to ~6% (Fig. 2B). Conversely, metabolism- and differentiation-related miRNAs such as let-7, miR-122, and miR-194 that represent the high abundant miRNAs in clusters IV and V [14, 22, 23], were promptly upregulated during development and reached a maximum level at the P7 and in adults (Fig. 2B). It is evident that the high abundant miRNAs could be divided into two categories: development-upregulated and development-downregulated miRNAs (Fig. 2C).

Super-enhancers are involved in regulating the expression of highly abundant and tissue-specific expression of miRNAs, which is critical for controlling cell identity [24–26], we, therefore, analyzed the super-enhancer signals during liver development by using H3K27ac ChIP-seq datasets [27]. As expected, a majority of highly expressed miRNAs were found to be driven by super-enhancers in a dynamic manner (Fig. 2D), which was consistent with the expression pattern from our HITS-CLIP data. For example, The H3K27ac signals of hematopoiesis-related miRNAs miR-142 and miR-144/miR-451 were very high at the early stages, but gradually decreased during development. While the signals of differentiation-related miRNAs such as let-7 and miR-122, increased and reached a maximum at the adult stage (Fig. 2E). Therefore, these analyses demonstrated that the super-enhancer signals coordinated with dynamic changes of high-abundance miRNA expression during development. We discovered that miR-122 was driven by the super-enhancer, providing a compelling reason for its tissue-specific and high-abundant expression in the adult mouse liver.

### Genome-wide identification and biological involvement analyses of miRNA targets during liver development

**Fig. 3:**
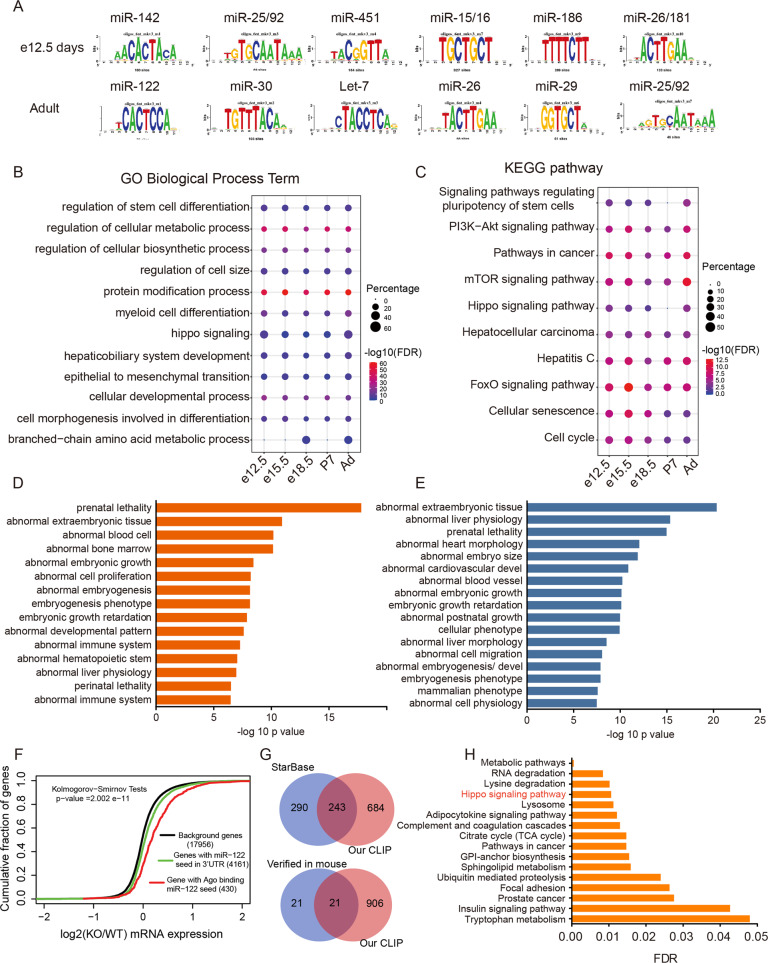
Genome-wide identification and biological involvement analyses of miRNA targets during liver development. **A** Motif analysis of Ago2-bound mRNAs representative of the developmental stages of liver. Up panel: e12.5, bottom panel: adult. **B** Representative of enriched Gene ontology (GO) biological process terms of targets of five clusters miRNAs. FDR false discovery rate; percentage, number of genes involved in a particular term divided by total genes in this term. **C** Representative of enriched KEGG pathways of targets of five clusters miRNAs. **D** Mammalian Phenotype (MP) Ontology enrichment analysis of targets of top 5 development-downregulated miRNAs. **E** MP Ontology enrichment analysis of targets of top 5 development-upregulated miRNAs. **F** Gene expression differences assessed by microarray analysis of background genes and targets predicted by seed in the 3′UTR or miR-122 between WT and miR-122 KO mice. 3′UTR targets identified by Ago HITS-CLIP. The gene expression data of WT and miR-122 KO mice were reported by Hsu et al. [15] and obtained from the GEO dataset (GSE20610). *p* values were calculated with a one-sided Kolmogorov−Smirnov test. **G** Venn diagram showing the overlap between mouse miR-122 targets identified by Starbase and our HITS-CLIP (up panel). Overlap between experimentally validated mouse miR-122 targets and our HITS-CLIP (bottom panel). The experimentally validated mouse miR-122 targets were obtained by integrating miRecords [64] and miRTarBase [65], two widely used databases collecting miRNA targets reported in the literature. **H** KEGG analysis of miR-122 targets identified by our Ago HITS-CLIP.

MiRNAs guide Ago proteins to bind to their target sites, the Ago binding sites of mRNAs should switch with the dynamic changes of the miRNA profile. Therefore, we analyzed the enriched motif of Ago-binding sites, and found the enriched motif of 3’UTR switched dynamically during development, which corresponded well to the change in the highly expressed miRNAs (Fig. 3A). For example, at e12.5, the most significantly enriched motifs matched miR-142 and miR-451 “seed regions”, while in the adult mouse liver, the motifs switched to the terminal differentiation-specific miRNAs miR-122, let-7.

Based on the miRNA-mRNA interactions, the functional in vivo targets of the five miRNA clusters were predicted (Table S3), which provides a genome-wide survey of miRNA targets correlated to the dynamic changes of miRNA expression during the liver development. GO and KEGG analysis of these targets showed significant enrichment of many processes that are crucial for liver development and function. Although targets of different miRNA clusters shared many important pathways or biological processes, dynamic changes of enrichment score were also observed at different stages (Fig. 3B, C).

We further analyzed the targets of the representatives of the classes of miRNAs that were highly expressed in early and late developmental stages (Table S4) by Mammalian Phenotype (MP) Ontology analysis [28]. Although all the miRNA targets were significantly enriched during embryo development processes, the development-downregulated miRNA targets were preferentially enriched in the development of blood cell and immune system (Fig. 3D), while the development-upregulated miRNA targets were involved in liver morphology and physiology (Fig. 3E).

Considering that miR-122 is liver-specific and is the most abundant miRNA in the late developmental stages of liver, we performed identification and confidence analysis of the miR-122 targets in more detail. We identified 927 miR-122 targets in 3’UTR, 1649 in CDS, and 340 in 5’UTR, respectively (Table S5). To test whether these targets are indeed functional in vivo, we first conducted integration analysis of the gene expression data of miR-122 knockout mice reported by Hsu et al. [15] with our HITS-CLIP. Because miRNAs can downregulate targets by destabilizing their mRNA [29, 30], changes in target mRNA levels were observed in most cases. In accordance with this, we observed that the genes harboring Ago-bound miR-122 sites had a significantly greater tendency to be upregulated than the background and purely seed-matched genes in miR-122 knockout mice (Fig. 3F). Moreover, the CDS targets of miR-122 also showed a significantly greater tendency to be upregulated than the background genes (Fig. S4A). Similar results were observed when analyzing miR-142 targets with gene expression of miR-142-knockout mouse T cells (GSE57543) (Fig. S4B, C). These assessments provided strong supports for the reliability of our miRNA targets from AGO HITS-CLIP.

Next, we compared our HITS-CLIP-identified miR-122 targets with those predicted by StarBase2.0 [31], approximately one-third of the targets showed overlap. We also compared HITS-CLIP-identified miR-122 targets with experimentally verified targets in mice (Fig. 3G). Among the 42 verified targets in mice, 21(50%) were identified in at least one stage of development (Fig. 3D and Table S6).

Our data from mouse liver development may provide an opportunity to test the functional miRNA targets by dynamic evidence collection. For example, Scarb1, a plasma membrane receptor of high density lipoprotein (HDL) cholesterol [32], is a novel miR-122 target identified in this study. The target site was first detected with very low peak at the early stage, while during the development, the peak gradually increased (Fig. S4D). This analysis not only provides dynamic evidence for the functional targets of miR-122, but also reveals that the regulation of cholesterol metabolism mainly occurred in the late developmental stages of liver.

We further performed GO and KEGG analysis on all the targets of miR-122 (Figs. 3H and S4E). As expected, metabolism is the most significant pathway regulated by miR-122 because the liver is the largest metabolic organ and critical for lipid, glutamine, and mitochondrial metabolism [16, 33, 34]. Interestingly, Hippo signaling pathway, which is critical for organ size control and tissue homeostasis in liver development [10, 11, 35], is among the most enriched pathways, highlighting the miR-122-mediated regulation of the Hippo pathway in liver development. We further performed pathway analysis of other development-upregulated miRNA targets, and found only miR-26a targets were also related to the Hippo pathway (Fig. S5), indicating a kind of cooperation with different miRNAs for the regulation of Hippo pathway in hepatocytes.

### miR-122 is a positive regulator of the Hippo pathway in liver cells

**Fig. 4:**
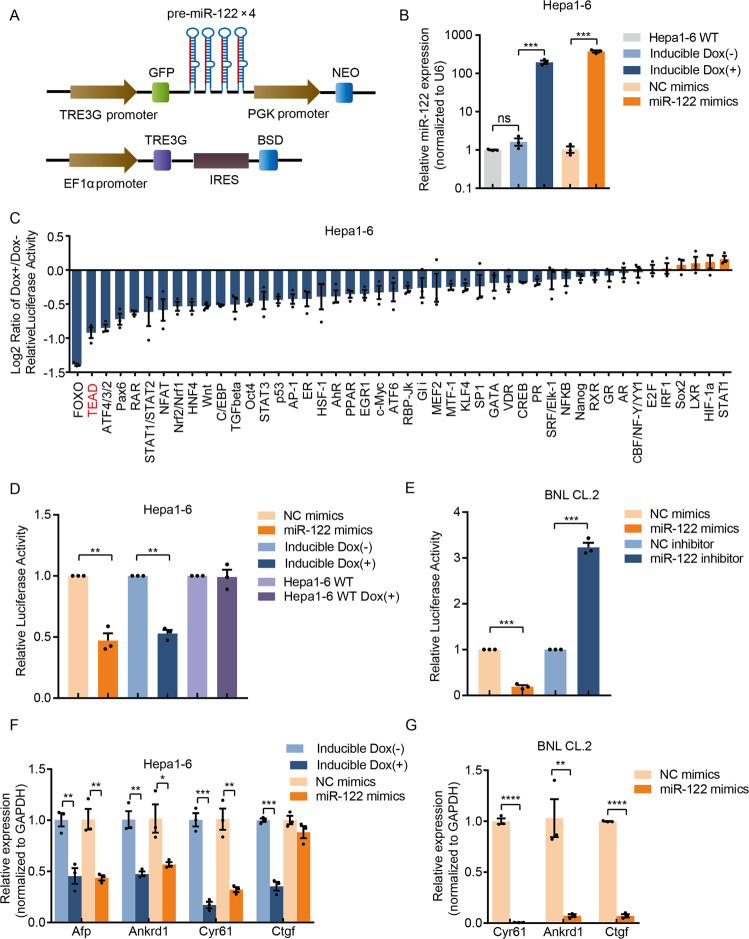
miR-122 positively regulates the Hippo pathway. **A** Structures of the inducible lentiviral plasmids expressing of miR-122. **B** Relative expression of miR-122 either after induced expression or transfection, values are mean ± SEM. **C** 46-pathway (Cignal 45-Pathway Reporter Array plus 8xGTIIC-luciferase Hippo pathway reporter) assays revealed the effects of miR-122 on the activity of various pathways in inducible Hepa1–6 cell lines. The data were log2-transformed, values are mean ± SEM. **D** 8xGTIIC-luciferase reporter assay of mimics transfection and inducible overexpression of miR-122; WT Hepa1–6 cells treated with doxycycline were also examined to rule out the influence of doxycycline, values are mean ± SEM. **E** 8xGTIIC-luciferase reporter assay after transfection of miR-122 mimics and inhibitors into BNL CL.2 cells, values are mean ± SEM. Analysis of the change of well-documented Hippo pathway downstream genes after either induced or mimics-transfection-mediated overexpression of miR-122 in Hepa1–6 cells (**F**) and BNL CL.2 cells (**G**), values are mean ± SEM. (*p < 0.05; **p < 0.01; ***p < 0.001; ****p < 0.0001).

To experimentally screen the pathways regulated by miR-122, we used a commercial pathway plasmid reporter system that allows the study of 45 important signaling pathways. As mentioned above, KEGG analysis indicated that miR-122 may regulate the Hippo pathway; therefore, we constructed a 8xGTIIC-luciferase reporter containing 8X YAP/TAZ-responsive synthetic TEAD binding sites [36]. Activation of Hippo signaling can be measured by evaluating the decrease of the transcriptional activities of TEAD, which is directly regulated by YAP and TAZ (Fig. S6B, C), two coactivators downstream of the Hippo pathway [37]. We also built inducible miR-122 overexpression cell lines to force miR-122 expression, miR-122 was overexpressed ~100-fold when doxycycline (Dox) was added (Figs. 4A, B and S6A).

Using pathway reporters and the miR-122-inducible expression cell lines, changes of 46 pathway activities were detected. Most of the pathways are inhibited by different degree, while a few of them are slightly activated by miR-122 (Fig. 4C). Remarkably, FOXO and TEAD, key regulators for lipid metabolism and cell proliferation, are identified as the top 2 among all the repressed pathways, which decreased to about 50% or more. It has been reported that miR-122 can suppress the FOXO pathway by targeting FOXO3 [38], but the regulation of the Hippo pathway by miR-122 was first identified in liver cells.

Furthermore, we used miR-122 mimics to overexpress miR-122, as expected, miR-122 mimics can sharply decrease the relative luciferase activities (Fig. 4D). Using luciferase reporter, the regulation of the Hippo pathway by miR-122 was also observed in human HCC cells by miR-122 mimics and inhibitors, which reveals that the regulation of the Hippo pathway by miR-122 is conserved among mouse and human (Fig. S6D, E). To further confirm the regulatory effect of miR-122 on the Hippo pathway, we analyzed the expression of four well-documented Hippo downstream genes that have no Ago-bound miR-122 seed, including *Ankrd1*, *Cyr61*, *Ctgf*, and *Afp* [39]. Using either miRNA mimics or inducible cell lines, forced expression of miR-122 resulted in significant decreases of these genes (Fig. 4F). Moreover, we overexpressed miR-122 in a mouse embryo liver cell line, BNL CL.2, and observed more significant increased activity of the Hippo pathway by both pathway reporter and RT-qPCR assays (Fig. 4E, G). We analyzed the microarray data of miR-122 KO mice from public dataset (GSE20610) [15]. We found the expression levels of many genes that are regulated by the Hippo pathway in liver [39], including *H19, Afp, Ctgf, Sox4, Cyr61 Ankrd1*, and *Birc2*, were increased when miR-122 was knocked out (Fig. S7). This data provides in vivo evidence that miR-122 regulates the Hippo pathway.

### miR-122 targets a number of hippo regulators including Taz and Ppp1cc

**Fig. 5:**
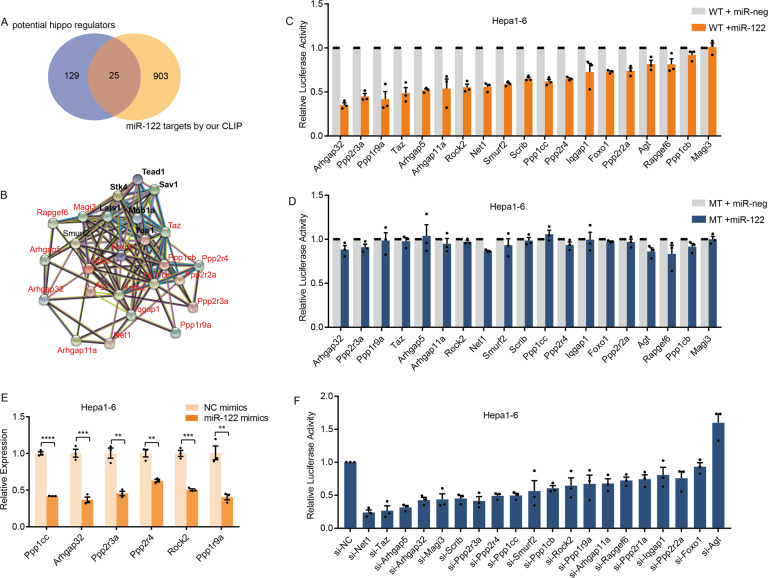
A number of miR-122 targets influence the Hippo pathway activity. **A** Veen diagram showing overlap between mouse miR-122 targets identified in this study and potential Hippo pathway regulators. The potential miR-122 targets were considered as Ago binding mRNA in 3′UTR, CDS with miR-122 seed sequences. **B** Network analysis of miR-122 targets that may serve as Hippo pathway regulators, the interactions between proteins were analyzed using string database. The miR-122 targets are indicated by red font, and the core component genes of Hippo pathway are indicated by black font. **C** miR-122 target wild-type dual-luciferase reporter assay, values are mean ± SEM. **D** miR-122 target mutant-type dual-luciferase reporter assay, values are mean ± SEM. **E** RT-qPCR showing the change of mRNA levels of targets after transfection of miR-122 mimics, values are mean ± SEM. **F** Hippo signaling pathway reporter assay after silencing potential miR-122 targets that may serve as regulator of Hippo pathway, values are mean ± SEM. (**p < 0.01; ***p < 0.001; ****p < 0.0001).

The core components of the Hippo pathway comprise a regulatory serine-threonine kinase module and a transcriptional module such as YAP and TAZ [40]. The Hippo pathway depends largely on kinase systems to control the phosphorylation of YAP and TAZ and their transcriptional activity. To further explore the mechanisms underlying miR-122 positive regulation of the Hippo pathway, we conducted integrated analysis of miR-122 targets with some studies that systemically identified regulators of the Hippo pathway [41]. Dozens of targets have been identified as candidates (Fig. 5A), all of them harbor classic target sites of miR-122 (Figs. S8, S9B, C), and network analysis revealed that they are in connection with core components of the Hippo pathway (Fig. 5B).

We validated the miRNA-targets interactions by dual-luciferase reporter assays in Hepa1–6 cells. Overexpression of miR-122 significantly repressed the luciferase activity of most candidate targets, while mutation of the seed region rescued the suppression (Fig. 5C, D). RT-qPCR showed the mRNA levels of six candidates were decreased significantly after miR-122 overexpression, revealing that the miR-122 can destabilize the mRNAs of these candidates (Fig. 5E).

To test the potential regulation on the Hippo pathway of these candidates, we silenced these genes by siRNAs pools and measured the activity of Hippo pathway by luciferase reporter. All the candidates were found to negatively influence the Hippo pathway activity with exception of Atg (Fig. 5F). It is worth noting that nine of them resulted in more than 50% decrease of luciferase activity when silenced by specific siRNAs.

**Fig. 6:**
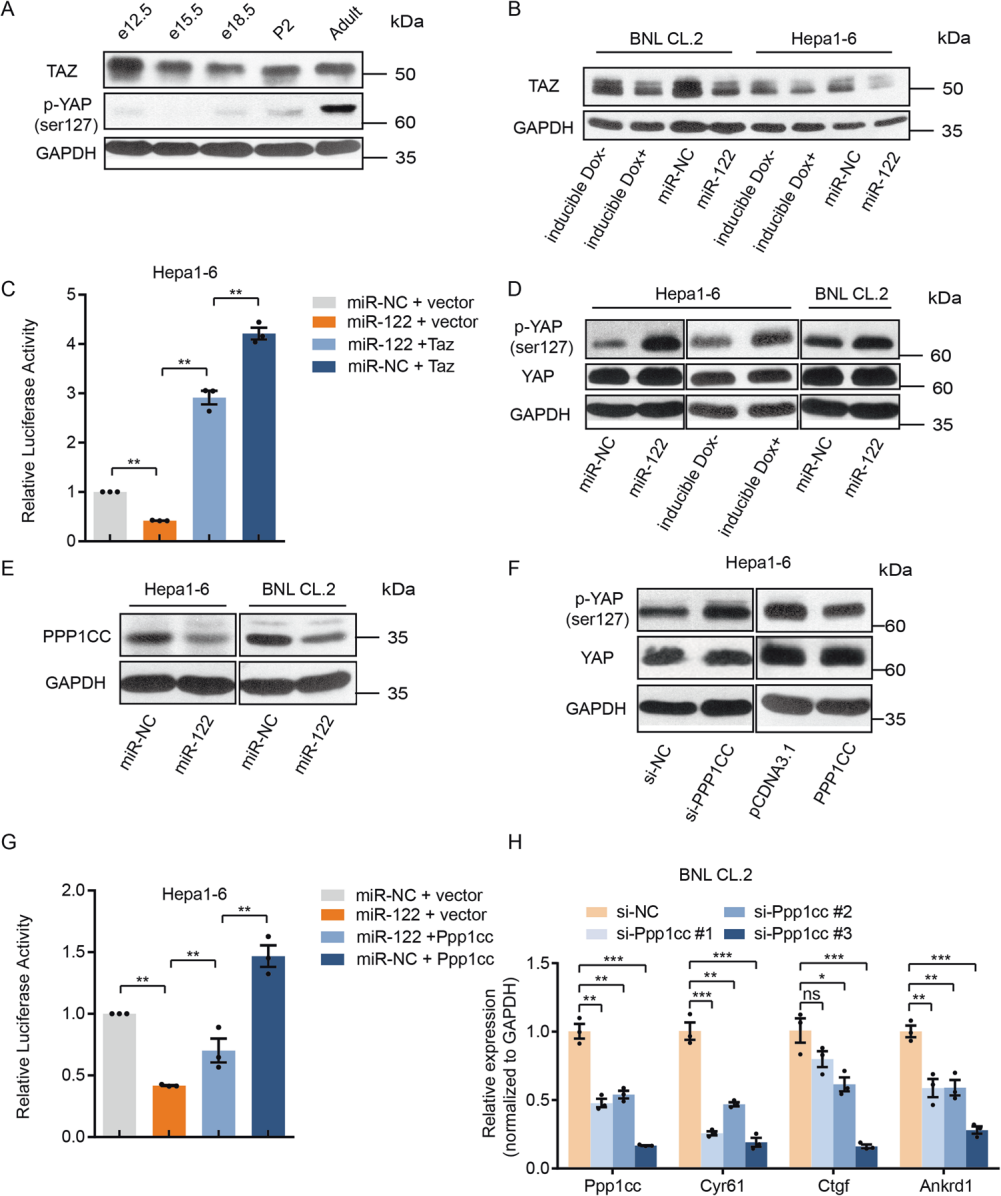
miR-122 can directly target Taz and promotes phosphorylation of YAP by targeting Ppp1cc. **A** Western blot showing the change of TAZ protein and YAP phosphorylated protein level during the mouse liver development. **B** Western blot showing the effect of overexpression of miR-122 on TAZ protein expression in Hepa1–6 and BNL CL.2 cells. **C** 8xGTIIC-luciferase reporter assay showing the effect of co-overexpression of miR-122 and Taz on the Hippo pathway, values are mean ± SEM. **D** Western blot showing the effect of overexpression of miR-122 on phosphorylation of YAP and the total YAP protein. **E** Western blot showing the effect of overexpression of miR-122 on PPP1CC protein expression in Hepa1–6 and BNL CL.2 cells. **F** Western blot showing the effect of knockdown (left) or overexpression (right) of Ppp1cc on phosphorylation of YAP. **G** 8xGTIIC-luciferase reporter assay showing the effect of co-overexpression of miR-122 and Ppp1cc on the Hippo pathway, values are mean ± SEM. **H** RT-qPCR showing the change of mRNA of Ppp1cc and hippo pathway downstream genes after silencing of Ppp1cc, values are mean ± SEM. (*p < 0.05; **p < 0.01; ***p < 0.001).

We first focused on Taz, which is core transcriptional co-factor of the Hippo signaling cascade [7, 8]. Western blot showed that the protein level of Taz decreased markedly at the e18.5 stage, and the abundance was maintained during liver development (Fig. 6A), which is negatively correlated with miR-122 expression. Overexpression of miR-122 by either mimics or two different inducible cell lines significantly reduced its protein level (Fig. 6B). In the rescue experiments by co-transfecting miR-122 and Taz, overexpression of Taz not only restored but also elevated the relative luciferase activity (Fig. 6C), which is reasonable given the greater intensity of overexpression than that of miR-122-mediated repression.

Since the Hippo pathway relies largely on kinase systems to control the phosphorylation of YAP and its transcriptional activity, we, therefore, detected phosphorylated YAP. Forced expression of miR-122 resulted in notably upregulated phosphorylated YAP but no detectable change in the total YAP in different liver cells (Fig. 6D). Moreover, the level of phosphorylation of YAP increased during liver development, which was positively correlated with miR-122 expression (Fig. 6A). To further explore the mechanism underlying miR-122-mediated promotion of YAP phosphorylation, we tested whether silencing of these candidate targets can result in the increase of YAP phosphorylation. We found silencing Ppp1cc, which encodes the catalytic subunit of PP1-gamma, caused a dramatic increase of YAP phosphorylation (Fig. S9A). Overexpression of Ppp1cc caused reduction of phosphorylation of YAP (Fig. 6F). Western blot showed the protein level of PPP1CC was significantly reduced when miR-122 mimics were transfected (Fig. 6E).

We further conducted a rescue experiment, restoring Ppp1cc in the miR-122-overexpressing cell line partially relieved the suppression of the transcriptional activity of TEAD (Fig. 6G). Moreover, RT-qPCR assays also showed silencing of Ppp1cc reduced the expression level of the Hippo pathway downstream genes (Fig. 6H).

Taken together, these results showed that miR-122 could positively regulate Hippo signaling by targeting numerous hippo regulators which constitute YAP1/TAZ-TEAD transcriptional networks.

## Discussion

**Fig. 7:**
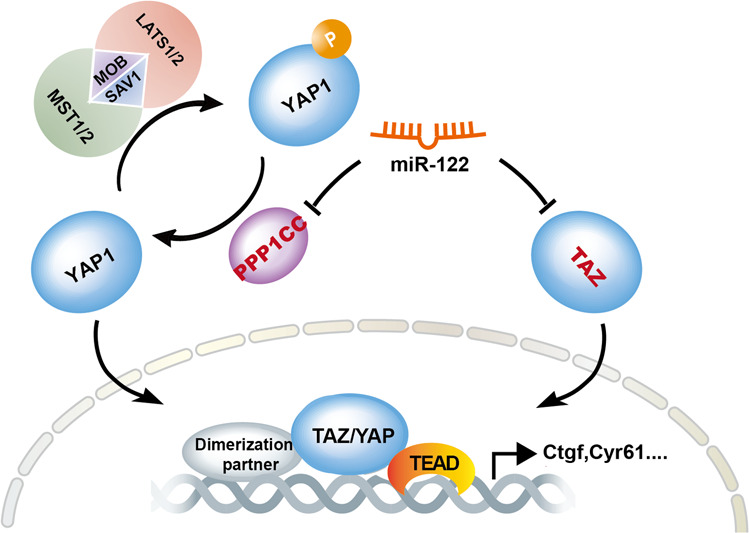
Working model showing miR-122 positively regulates the Hippo pathway by targeting hippo regulators. Remarkably, on one hand, miR-122 can directly target the transcription co-activator Taz, on the other hand, miR-122 can influence phosphorylation of YAP through targeting Ppp1cc.

Liver-specific miR-122 has emerged as a critical regulator of multiple processes in hepatocytes [12, 14, 42–44]. By integrating analysis of the targetome obtained by AGO HITS-CLIP and pathway reporter assays, our analysis reveals a new function of miR-122, which serves as a key regulator of the Hippo pathway. Mechanistically, miR-122 negatively regulated the outcomes of the Hippo pathway transcription factor TEAD by targeting a number of hippo regulators including TAZ and phosphatases subunit PPP1CC that dephosphorylates YAP (Fig. 7). Our study reveals a new regulatory network which miR-122 and the Hippo pathway synergistically regulate the terminal differentiation of hepatocytes. Considering miRNA usually have multiple targets to synergistically regulate biological processes, miR-122 may also indirectly regulate the Hippo pathway by targeting genes that is complementary to the Hippo pathway.

Previous studies of miR-122 knockout mice showed phenotypes highlighted by hepatic steatosis, liver inflammation, fibrosis, and HCC [15, 45, 46]. In these mice, the lipid metabolism disorder was caused by direct effects of miR-122 deficiency, and eventually resulted in liver inflammation and fibrosis [15, 16]. However, the mechanism underlying a high incidence of HCC in miR-122 knockout mice was not fully resolved. We identified miR-122 as an important regulator for both Hippo and FOXO pathways in hepatocytes. As the two pathways have been reported as key regulators of cancers, inflammation, and lipid metabolism [9, 47–50], loss of miR-122 may lead to activate YAP/TAZ-TEAD transcriptional networks that promote cell proliferation and largely increase the liver tumor incidence driven by a chronic aseptic inflammation.

We also identified a set of high-abundance and development-upregulated miRNAs such as let-7, miR-16, miR-194, miR-126, miR-30, and miR-26, in the late developmental stages of the mouse liver, as well as their targetomes during the terminal differentiation of hepatocytes (Fig. 2B and Table S3). Although these miRNAs are not liver-specific, they are related to the mature epithelial cells of various tissues, indicating that they may be a group of development-upregulated miRNAs required for promoting and maintaining terminal differentiation of hepatocytes and other epithelial cells. In particular, it is worth mentioning that all of these miRNAs have been reported as important tumor suppressors and/or epithelial-mesenchymal transition modulators [51–53], which is consistent with our targetome analyses of these miRNAs (Fig. S5), indicating the strong inverse correlation between the development-upregulated miRNAs and the occurrence of hepatocellular carcinoma and other cancers.

In contrast to the abovementioned miRNAs, we also identified a set of high-abundance, development-downregulated miRNAs with their targetomes during the early developmental stages of the mouse liver (Fig. 2B and Table S3), which are evidently hematopoietic and stem-cell related. The dynamic changes of these miRNAs is consistent with the fact that fetal liver is the major hematopoietic organ during embryogenesis [54, 55], and the hematopoietic cells and embryonic hepatocytes provide indispensable environments for each other [56, 57]. Our study identified the targetome of these miRNAs, which provides valuable information for studies of these miRNAs in the fetal liver.

Overall, we conducted comprehensive analyses of the dynamic changes of miRNAs and their targetomes during the liver development (Table S7). Our study demonstrated miR-122 regulates the Hippo pathway by targeting multiple genes including Taz and Ppp1cc, providing new insights into the function of miR-122 in liver development and pathology. Our data also reveals that a large number of miRNAs with dynamic abundance participate in the development of liver in a spatiotemporal manner. Considering that each miRNA may have multiple targets, the targetomes of all miRNAs obtained by this study provide better insights into the regulatory networks of the interplay among miRNAs, transcriptional factors, and epigenetic regulators that are involved in various signaling pathways.

## Materials and methods

### Animals and cells

C57BL/6J adult mice and pregnant mice were provided by the laboratory animal center of Sun Yat-sen University. Fetal (e12.5, e15.5, and e18.5), newborn (P7), and adult (6–8 weeks) mouse livers were used, mice from the same developmental stages were chosen randomly. The day the mice exhibited vaginal plugs after mating was considered 0.5 days (e0.5).

The Hepa1–6, BNL CL.2, HepG2, Huh7, and 293T cell lines used were obtained from ATCC and were cultured in Dulbecco’s Modified Eagle Medium (DMEM) supplemented with 10% FBS at 37 °C and 5% CO2. The miR-122-Tet-On Hepa1–6, BNL CL.2 stable cell line was generated using lentiviral vectors as we described previously [58].

### siRNAs and miRNA mimics

miR-122 mimics, Yap Taz siRNAs were purchased from Genepharma (Shanghai, China), and the sequence is listed in Table S8. Ppp1cc and other gene siRNA pools for screening candidate regulating phosphorylation of YAP were purchased from RiboBio (Guangzhou, China).

### Vector construction and luciferase reporter assays

The Cignal Finder 45-Pathway Reporter were purchased from Qiagen, and the 8xGTIIC-luciferase Hippo pathway reporter were generated as reported [36]. As described previously [14], dual-luciferase reporter vectors for miRNA target sites were constructed based on psiCHECK-2. Briefly, 47-nt DNA fragments containing the putative binding site for miRNA of each target site (~30-nt of 5′ flanking sequences, seed-matched sequences, 10-nt of 3’ flanking sequences) were cloned into the psiCHECK-2 vector using Xho I/NotI restriction enzyme.

Hepa1–6 or BNL CL.2 cells were transfected with 20 nM siRNAs, miRNAs, or 100 ng plasmid in 96-well plate using Lipofectamine^TM^ 2000 (Invitrogen). After 2 days of culture, the cells were lysed in passive lysis buffer and luciferase activities were measured with a Dual Luciferase Assay Kit (Promega).

### Real-time quantitative PCR (RT-qPCR)

Cells were grown and treated as described above, and then, RNA was collected using TRIzol Reagent (Invitrogen), RNA was reverse-transcribed using the PrimeScript RT Reagent Kit (Takara RR047A) and real-time PCR was performed using SYBR Premix ExTaq™ (Takara) analyzed by StepOne™ real-time PCR instrument (ABI), The primers are listed in Table S8. The expression was first compared with that of an endogenous control (GAPDH or U6), and the normalized values were subjected to the comparative Ct (^ΔΔ^CT) analysis method to calculate the fold change between the control and experimental groups.

### Western blot

Cells or homogenized tissue were lysed in ice-cold RIPA buffer supplemented with protease inhibitor cocktail (Roche, 4693132001) and phosphatase inhibitor (Roche, 4906837001). A BCA Protein Assay Kit (Thermo Fisher) was used to measure the concentrations of the protein samples. Equal amounts of protein samples were separated by SDS gel electrophoresis, and then were transferred to nitrocellulose membrane. Then, the membranes were blocked with 5% milk for 1 h. Primary antibodies were incubated overnight at 4 °C and horseradish peroxidase-conjugated secondary antibodies were used to bind the primary antibodies. Chemiluminescent HRP Substrate (Millipore, WBKLS0500) was used to visualize the signal of proteins. The primary antibodies used were as follows: Ago2 (Wako, #018–22021 and Abnova, #H00027161-M01), Ago1 (MBL, #RN028PW), and PPP1CC (Proteintech Cat# 55150-1-AP). β-Actin (#4970), GAPDH (# 2118), YAP (#14074), YAP/TAZ (#8418), p-YAP (Ser127, #13008), p-YAP (Ser397, #13619) were purchased from Cell Signaling Technology.

### Ago2 HITS-CLIP and data analysis

The detailed Ago2 HITS-CLIP experiments were performed as previously reported [18] with some modifications. The Ago2 HITS-CLIP were conducted in two biological replicates. The embryonic or adult mouse livers were washed dissected, homogenized, and crosslinked 3 times using 254 nm UV light (0.2 J/cm^2^) in cold PBS. The tissue were washed once with PBS and lysed with lysis buffer. The lysates were first precleared with 150 μL of Protein G Dynabeads and then incubated with Ago2 antibodies (Wako, #018-22021 and Abnova, #H00027161-M01 15 μg Ab of each), and 300 μL of Protein G Dynabeads for each reaction overnight at 4 °C. For P7 and Adult mouse liver, Ago1 antibodies (MBL, #RN028PW) were also added. The Ago-binding RNAs were then dephosphorylated using alkaline phosphatase (Fermentas, EF0651), and then, the 3’ adapter was ligated on-bead by truncated T4 RNA ligase 2 (NEB, M0242). PNK treatment was then conducted (first with 32 P-γ-ATP and then cold ATP). The samples were separated with SDS-PAGE and then transferred to a nitrocellulose membrane. According to the autoradiogram signal, the Ago-RNA complex regions were excised from the membrane and digested with proteinase K. RNA was isolated and ligated with 5’ RNA adapters and then reverse transcribed to cDNAs. After optimizing the best amplification cycles by RT-qPCR, the cDNA were amplified and PCR product were sent for high-throughput sequencing. Adaptors and primers are listed in Table S8.

For data analysis, FASTX-Toolkit was used to preprocess the HITS-CLIP raw data and Bowtie tools were used for mapping the sequences into the mouse genome (mm10) and miRNA precursor (mirBase v19). Overlapping reads were grouped to identify clusters. The clusters were then annotated according to the UCSC refgene and Ensembl annotations. The clusters sequences of mRNA were scanned to identify miRNA-target interactions by matching the seed sequences of all known mouse miRNAs. GO and KEGG analysis was performed using DAVID [59] and ConsensusPathDB tools[60]. The motif of Ago-binding sites were analyzed using RSAT tools [61]

### H3k27ac ChIP-seq data analysis

Mouse liver H3k27ac ChIP-seq data were downloaded from GEO database (GSE52386). The sequences of ChIP-seq were aligned to the mouse genome (mm10) using BOWTIE [62]. The peaks were generated using MACS and the super-enhancers were analyzed using ROSE [63] software. The SEs were then assigned to the nearest TSS of gene or miRNAs with ± 100 kb.

### Statistical analysis

All data were shown as mean ± standard error of mean (SME) processed by GraphPad 6 (GraphPad Software Inc.). Student’s t-test was used to test for statistical significance of the differences between two different group parameters and One-Way ANOVA followed by Bonferroni test was used for multiple comparisons. One-sided Kolmogorov−Smirnov test was used for analyzing the cumulative frequency curve. *p* values < 0.05 were considered statistically significant (*p < 0.05; **p < 0.01; ***p < 0.001; ****p < 0.0001).

## Supplementary information

Supplementary Figures

Table S1

Table S2

Table S3

Table S4

Table S5

Table S6

Table S7

Table S8

Reproducibility checklist

## Data Availability

Ago HITS-CLIP data in this study have been deposited in the NCBI’s Gene GEO under accession code GSE153876.
