# Supplementary material for: Genome-wide identification of microRNA targets reveals positive regulation of the Hippo pathway by miR-122 during liver development
Source: Cell Death Dis. 2021 Dec 14;12(12):1161. doi: 10.1038/s41419-021-04436-7 (PMC8671590; doi:10.1038/s41419-021-04436-7)
Supplement: Supplementary file 1 — Supplementary Figures [file 41419_2021_4436_MOESM1_ESM.pdf]

Titles and Legends to Supplementary Figures

Figure S1: HITS-CLIP Antibody verification and AGO expression in mouse liver during development

(A, B, C) Detection of the specificity of AGO antibodies by Western blot from Wako (A), Abnova (B) or MBL (C).

(D, E, F) Dectection of antibodies by silver stain (D),and immunoprecipitation (E,F).

(G, H) Detection of mRNA (G) and protein (H) expression of Ago among different development stages of mouse liver.

Figure S2: The crosslinking and digestion conditions of HITS-CLIP were optimized to obtain high quality and suitable length products

(A) Detection of Uv cross-linking efficiency by autoradiogram.

(B) Detection of RNase digestion by autoradiogram.

(C, D) Detection of PCR product by PAGE electrophoresis (C) and Agilent 2100(D) analysis.

Figure S3: Analysis of AGO-bound miRNA expression profile of mouse liver from various developmental stages

(A) Length distribution of fragment produced by HITS-CLIP.

(B) Correlation analysis of miRNA expression among different developmental stages (log2 normalized miRNA reads)

(C) Genome location distribution of Ago binding RNA reads.

Figure S4: Analysis of miRNA targets identified by HITS-CLIP

(A) Gene expression differences assessed by microarray of background genes, or

miR-122 targets identified by Ago HITS-CLIP in the coding region between WT and miR-122 KO mice, p values were calculated with a one-sided Kolmogorov-Smirnov test.

(B, C) Cumulative distribution curve of target mRNA level change after miR-142 knock out (B) 3'UTR targets and (C) CDS targets. The gene expression data of WT and miR-142 KO mice T cells were obtained from the GEO dataset (GSE20610). p values were calculated with a one-sided Kolmogorov-Smirnov test.

(D) IGV showed the change of Ago binding miR-122 targeting site of the Scarb1 gene during mouse liver development.

(E) Gene Ontology (GO) analysis of miR-122 targetome.

Figure S5: KEGG pathway analysis of targets of development-upregulated miRNAs with high abundance

Figure S6: The establishment of inducible overexpression miR-122 cell line and Hippo pathway reporter plasmid

(A) Change of GFP fluorescence before and after inducible expressing miR-122 in Hepa1-6 cell line.

(B) Schematic diagram of the skeleton of Hippo pathway reporter plasmid.

(C) Detection of the effectiveness 8xGTIIC-luciferase Hippo pathway reporter in and HeLa cell lines by YAP siRNAs, values are mean ± SEM. (D) The effect of miR-122 mimics on Hippo pathway activities, which was measured by 8xGTIIC-luciferase reporter in HepG2 cells. (E) The effect of miR-122 inhibitors on Hippo pathway activities, which was measured by 8xGTIIC-luciferase reporter in Huh7 cells.

Figure 7: The expression the Hippo pathway downstream genes in miR-122 KO

mice liver. Public microarray dataset (GSE20610) of miR-122 KO mice were analyzed to detect the influence of miR-122 on genes Hippo pathway-target genes *in vivo*.

Figure 8: Predicted miR-122 binding sites of the targets. The putative miR-122 target base pair interactions were predicted by miRanda.

Figure 9: influence of candidate targets on phosphorylation of YAP. (A). Western Blot showing the impact of silencing candidate genes on phosphorylation of YAP. (B) Ago HITS-CLIP showing the miR-122 target site at the 3’UTR of Taz (Wwtr1). (C) Ago HITS-CLIP showing the miR-122 target site at the 3’UTR of Ppp1cc.

Figure S1

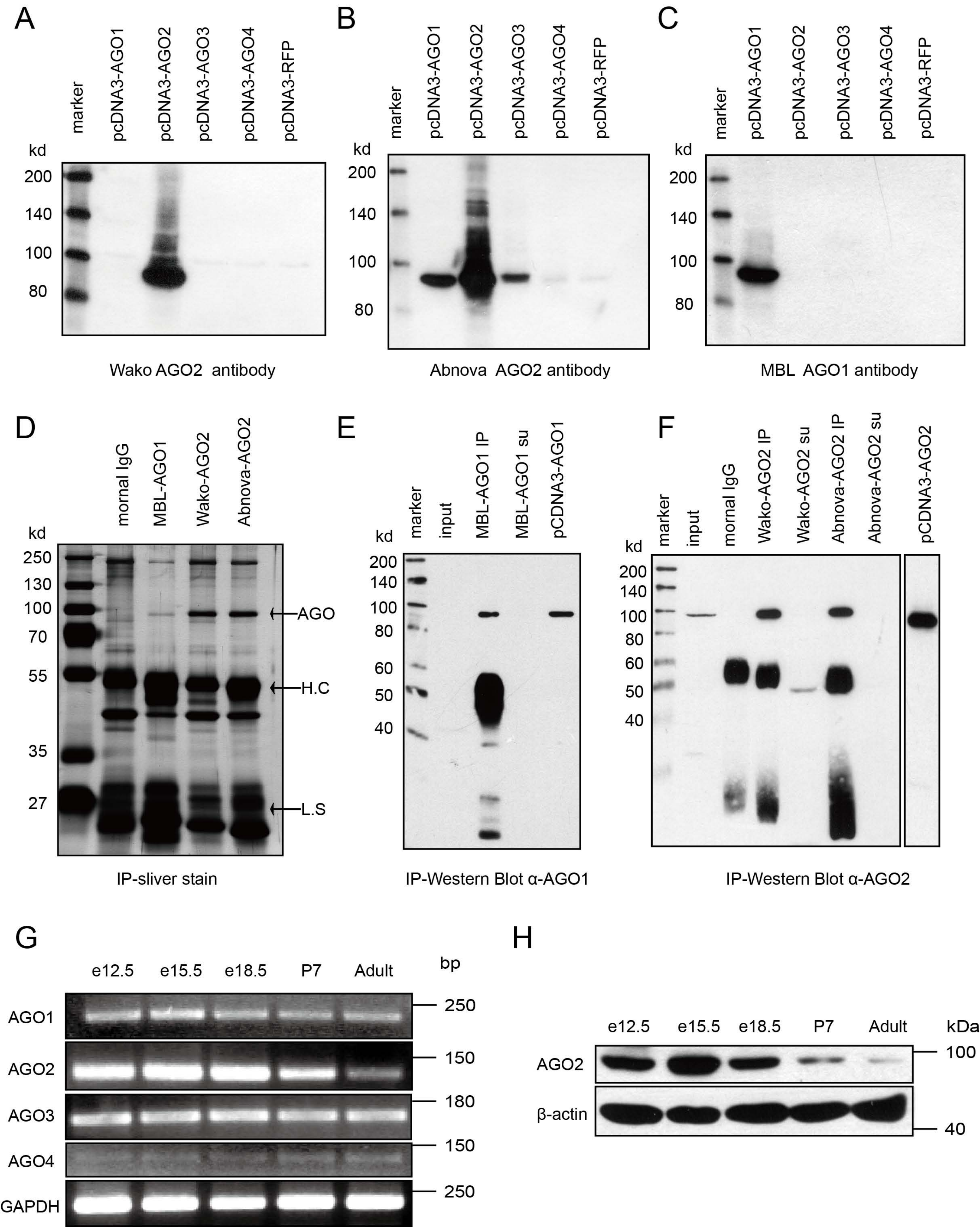

A
marker
pcDNA3-AGO1
pcDNA3-AGO2
pcDNA3-AGO3
pcDNA3-AGO4
pcDNA3-RFP
kd
200
140
100
80
Wako AGO2 antibody
B
marker
pcDNA3-AGO1
pcDNA3-AGO2
pcDNA3-AGO3
pcDNA3-AGO4
pcDNA3-RFP
kd
200
140
100
80
Abnova AGO2 antibody
C
marker
pcDNA3-AGO1
pcDNA3-AGO2
pcDNA3-AGO3
pcDNA3-AGO4
pcDNA3-RFP
kd
200
140
100
80
MBL AGO1 antibody
D
mornal IgG
MBL-AGO1
Wako-AGO2
Abnova-AGO2
kd
250
130
100
70
55
35
27
AGO
H.C
L.S
IP-sliver stain
E
marker
input
MBL-AGO1 IP
MBL-AGO1 su
pCDNA3-AGO1
kd
200
140
100
80
60
50
40
IP-Western Blot α-AGO1
F
marker
input
mornal IgG
Wako-AGO2 IP
Wako-AGO2 su
Abnova-AGO2 IP
Abnova-AGO2 su
pCDNA3-AGO2
kd
200
140
100
80
60
50
40
IP-Western Blot α-AGO2
G
e12.5
e15.5
e18.5
P7
Adult
bp
AGO1
AGO2
AGO3
AGO4
GAPDH
250
150
180
150
250
H
e12.5
e15.5
e18.5
P7
Adult
kDa
AGO2
β-actin
100
40

Figure S2

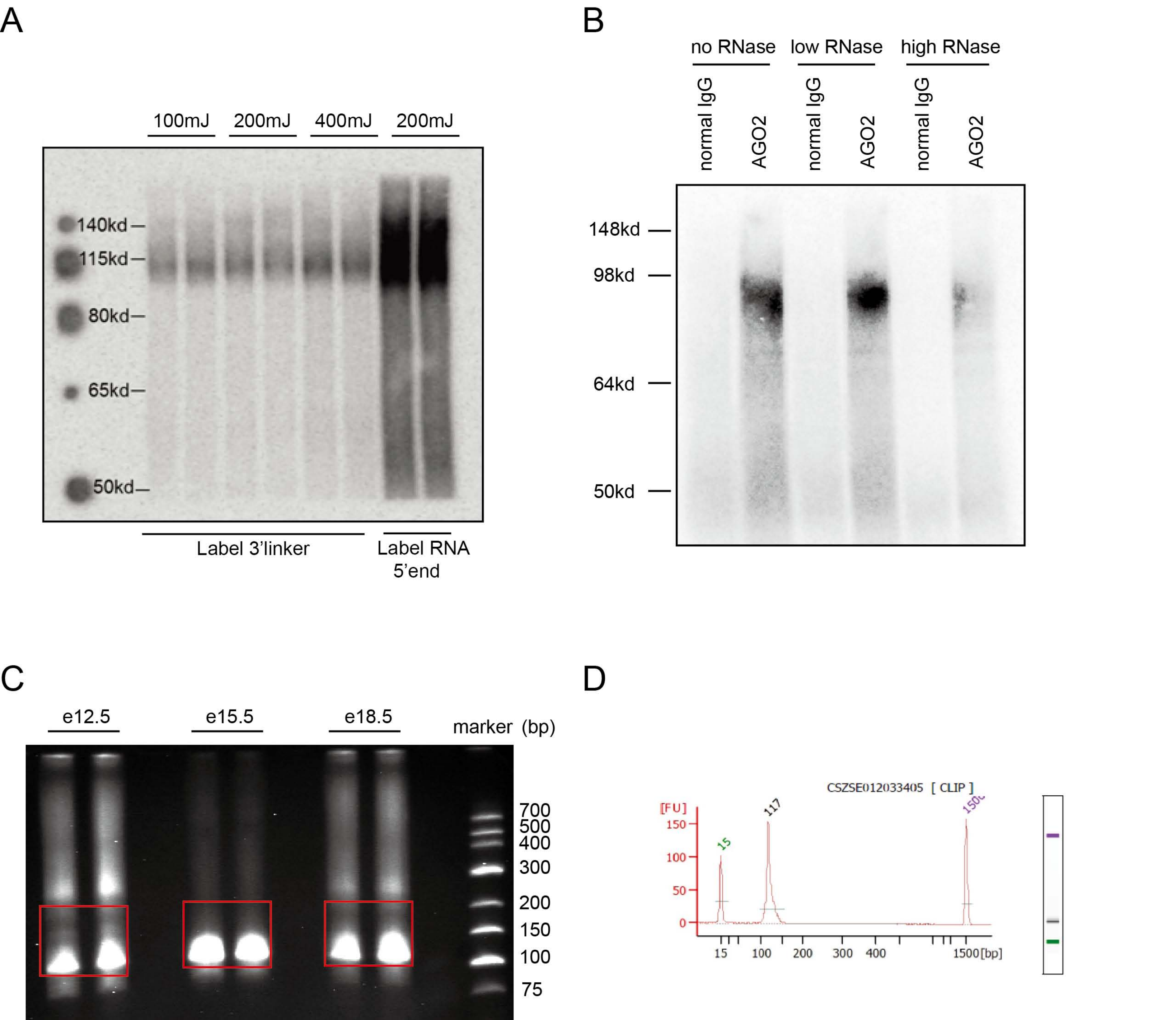
A
100mJ
200mJ
400mJ
200mJ
140kd
115kd
80kd
65kd
50kd
Label 3'linker
Label RNA 5'end
B
no RNase
low RNase
high RNase
normal IgG
AGO2
normal IgG
AGO2
normal IgG
AGO2
148kd
98kd
64kd
50kd
C
e12.5
e15.5
e18.5
marker (bp)
700
500
400
300
200
150
100
75
D
CSZSE012033405 [CLIP]
[FU]
150
100
50
0
15
117
15
100
200
300
400
1500 [bp]

Figure S3

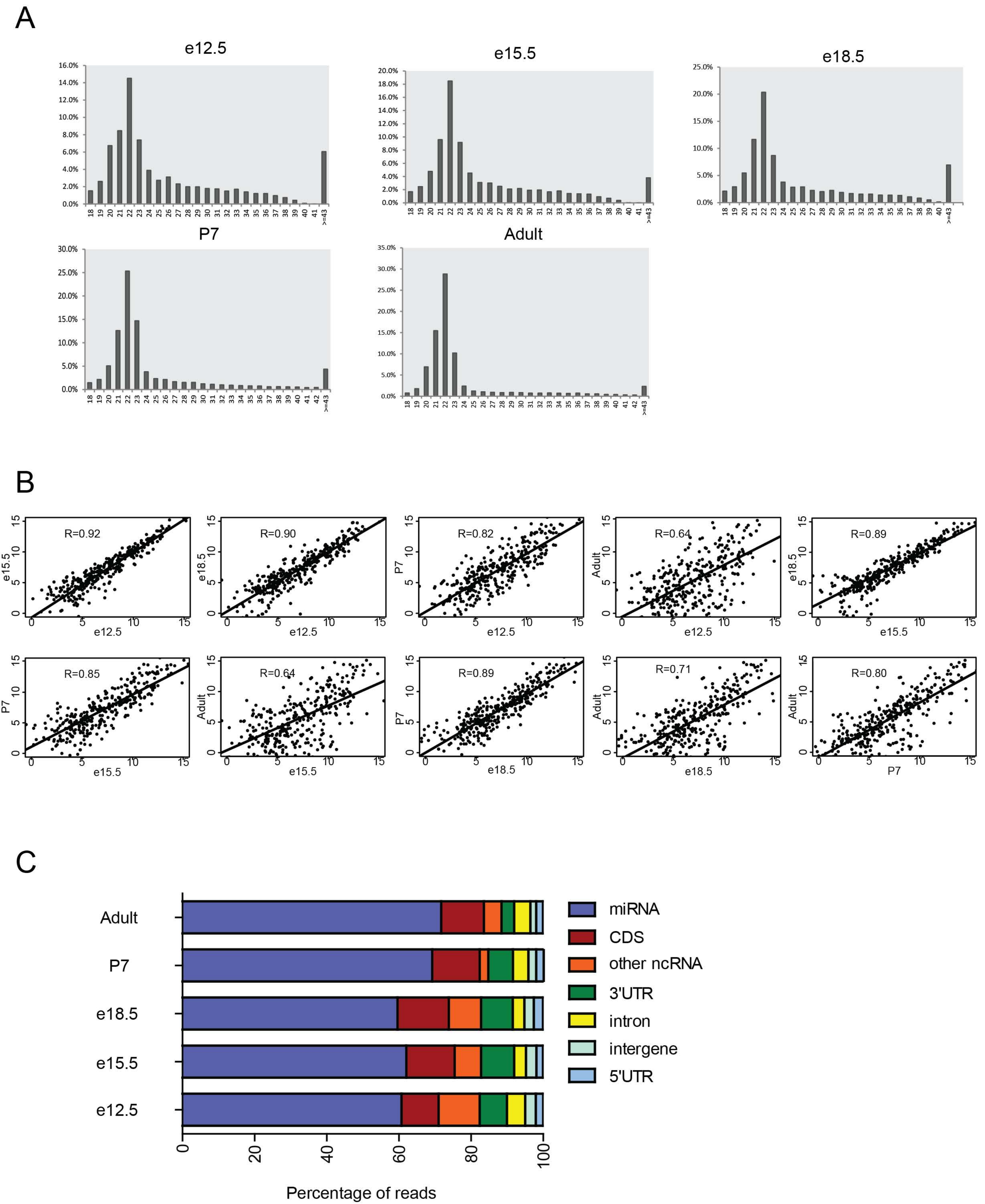
A
e12.5
e15.5
e18.5
P7
Adult
B
R=0.92
R=0.90
R=0.82
R=0.64
R=0.89
R=0.85
R=0.64
R=0.89
R=0.71
R=0.80
e12.5
e15.5
e18.5
P7
Adult
C
Adult
P7
e18.5
e15.5
e12.5
miRNA
CDS
other ncRNA
3'UTR
intron
intergene
5'UTR
Percentage of reads

Figure S4

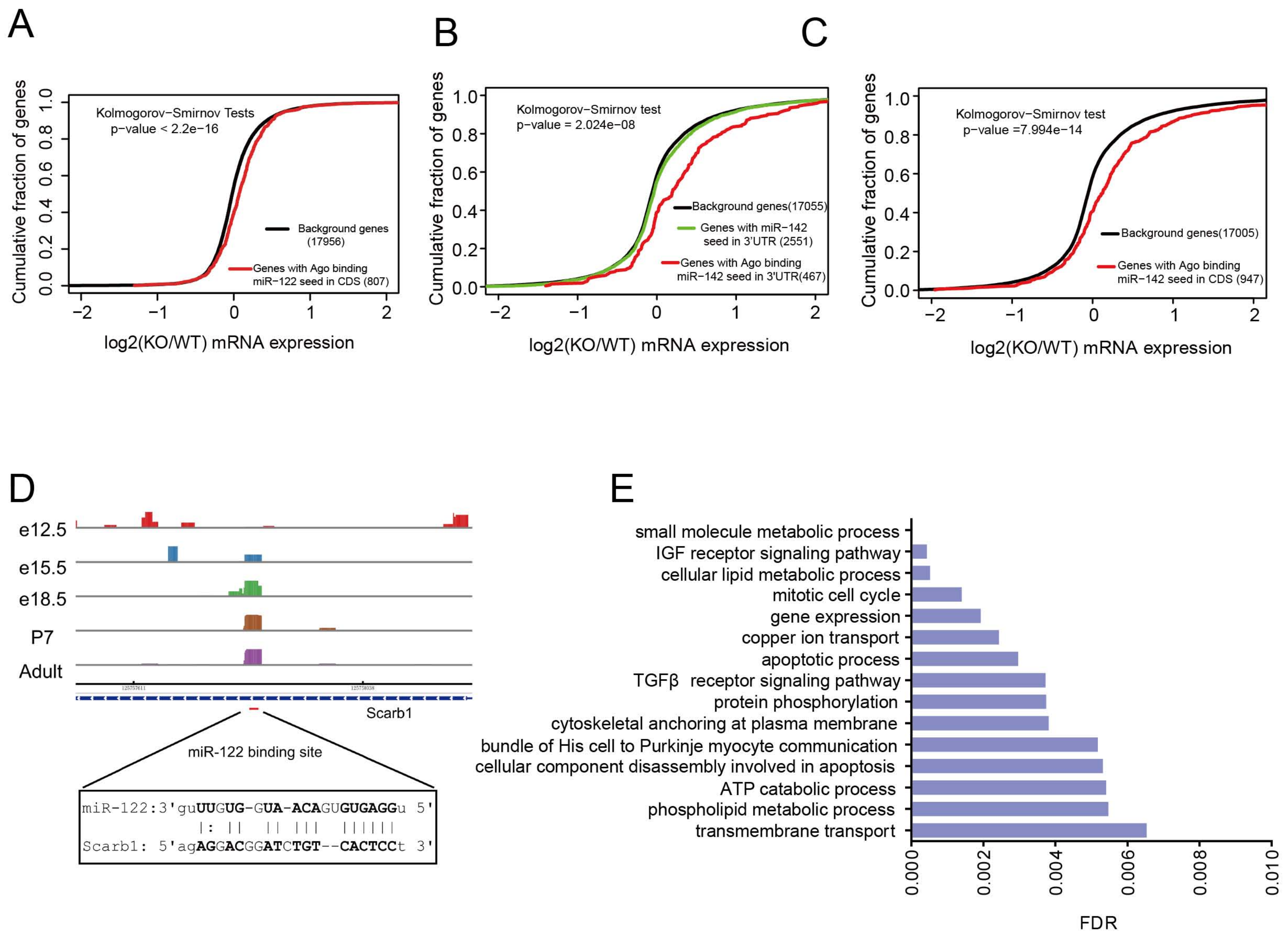
A
Kolmogorov−Smirnov Tests
p−value < 2.2e−16
Background genes (17956)
Genes with Ago binding miR−122 seed in CDS (807)
Cumulative fraction of genes
log2(KO/WT) mRNA expression
B
Kolmogorov−Smirnov test
p−value = 2.024e−08
Background genes(17055)
Genes with miR−142 seed in 3'UTR (2551)
Genes with Ago binding miR−142 seed in 3'UTR(467)
Cumulative fraction of genes
log2(KO/WT) mRNA expression
C
Kolmogorov−Smirnov test
p−value =7.994e−14
Background genes(17005)
Genes with Ago binding miR−142 seed in CDS (947)
Cumulative fraction of genes
log2(KO/WT) mRNA expression
D
e12.5
e15.5
e18.5
P7
Adult
Scarb1
miR-122 binding site
miR-122:3'guUUGUG-GUA-ACAGUGUGAGGu 5'
Scarb1: 5'agAGGACGGATCTGT--CACTCCt 3'
E
small molecule metabolic process
IGF receptor signaling pathway
cellular lipid metabolic process
mitotic cell cycle
gene expression
copper ion transport
apoptotic process
TGFβ receptor signaling pathway
protein phosphorylation
cytoskeletal anchoring at plasma membrane
bundle of His cell to Purkinje myocyte communication
cellular component disassembly involved in apoptosis
ATP catabolic process
phospholipid metabolic process
transmembrane transport
FDR

Figure S5

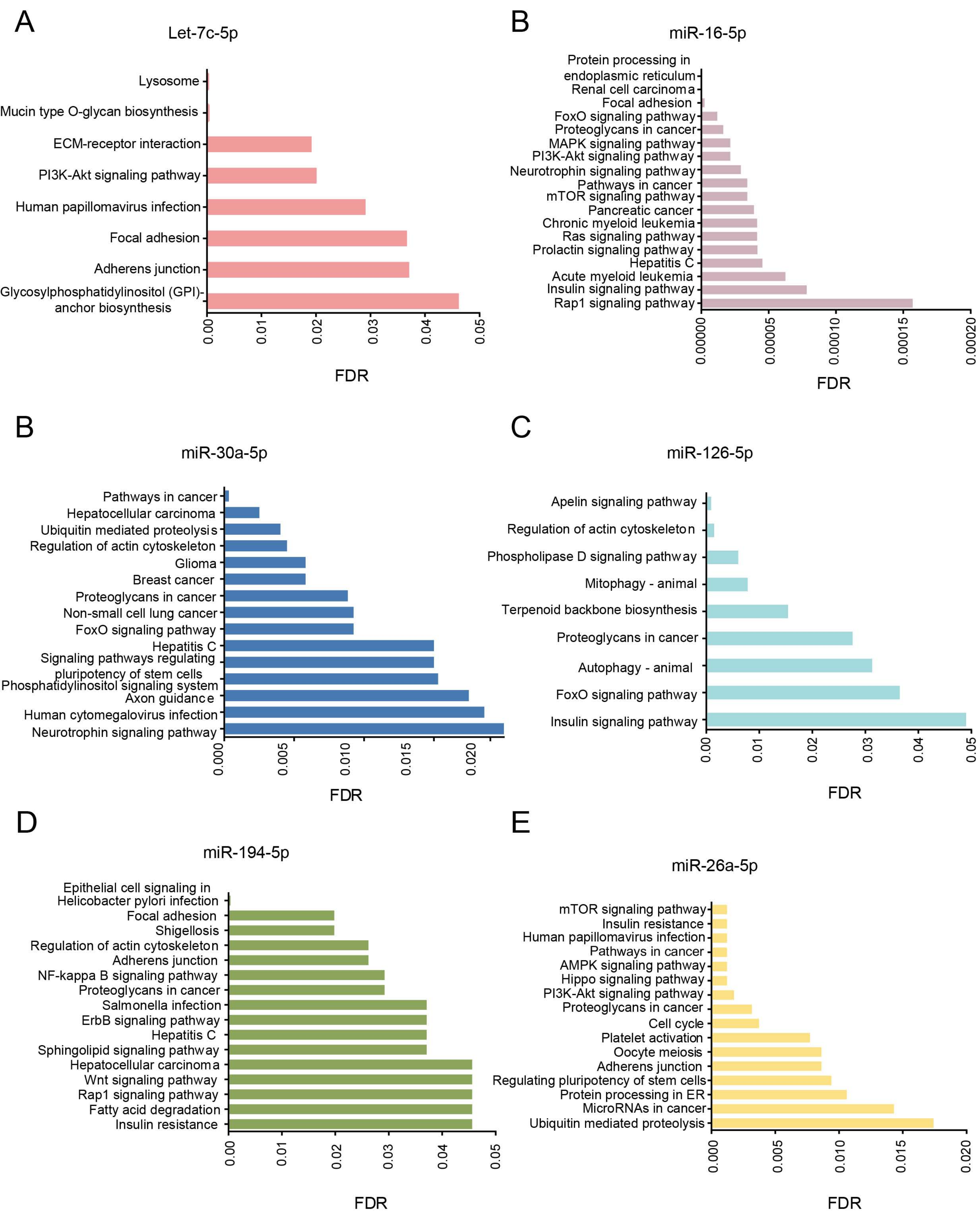

A
Let-7c-5p
Lysosome
Mucin type O-glycan biosynthesis
ECM-receptor interaction
PI3K-Akt signaling pathway
Human papillomavirus infection
Focal adhesion
Adherens junction
Glycosylphosphatidylinositol (GPI)-anchor biosynthesis
0.00
0.01
0.02
0.03
0.04
0.05
FDR
B
miR-16-5p
Protein processing in endoplasmic reticulum
Renal cell carcinoma
Focal adhesion
FoxO signaling pathway
Proteoglycans in cancer
MAPK signaling pathway
PI3K-Akt signaling pathway
Neurotrophin signaling pathway
Pathways in cancer
mTOR signaling pathway
Pancreatic cancer
Chronic myeloid leukemia
Ras signaling pathway
Prolactin signaling pathway
Hepatitis C
Acute myeloid leukemia
Insulin signaling pathway
Rap1 signaling pathway
0.00000
0.00005
0.00010
0.00015
0.00020
FDR
B
miR-30a-5p
Pathways in cancer
Hepatocellular carcinoma
Ubiquitin mediated proteolysis
Regulation of actin cytoskeleton
Glioma
Breast cancer
Proteoglycans in cancer
Non-small cell lung cancer
FoxO signaling pathway
Hepatitis C
Signaling pathways regulating pluripotency of stem cells
Phosphatidylinositol signaling system
Axon guidance
Human cytomegalovirus infection
Neurotrophin signaling pathway
0.000
0.005
0.010
0.015
0.020
FDR
C
miR-126-5p
Apelin signaling pathway
Regulation of actin cytoskeleton
Phospholipase D signaling pathway
Mitophagy - animal
Terpenoid backbone biosynthesis
Proteoglycans in cancer
Autophagy - animal
FoxO signaling pathway
Insulin signaling pathway
0.00
0.01
0.02
0.03
0.04
0.05
FDR
D
miR-194-5p
Epithelial cell signaling in Helicobacter pylori infection
Focal adhesion
Shigellosis
Regulation of actin cytoskeleton
Adherens junction
NF-kappa B signaling pathway
Proteoglycans in cancer
Salmonella infection
ErbB signaling pathway
Hepatitis C
Sphingolipid signaling pathway
Hepatocellular carcinoma
Wnt signaling pathway
Rap1 signaling pathway
Fatty acid degradation
Insulin resistance
0.00
0.01
0.02
0.03
0.04
0.05
FDR
E
miR-26a-5p
mTOR signaling pathway
Insulin resistance
Human papillomavirus infection
Pathways in cancer
AMPK signaling pathway
Hippo signaling pathway
PI3K-Akt signaling pathway
Proteoglycans in cancer
Cell cycle
Platelet activation
Oocyte meiosis
Adherens junction
Regulating pluripotency of stem cells
Protein processing in ER
MicroRNAs in cancer
Ubiquitin mediated proteolysis
0.000
0.005
0.010
0.015
0.020
FDR

Figure S6

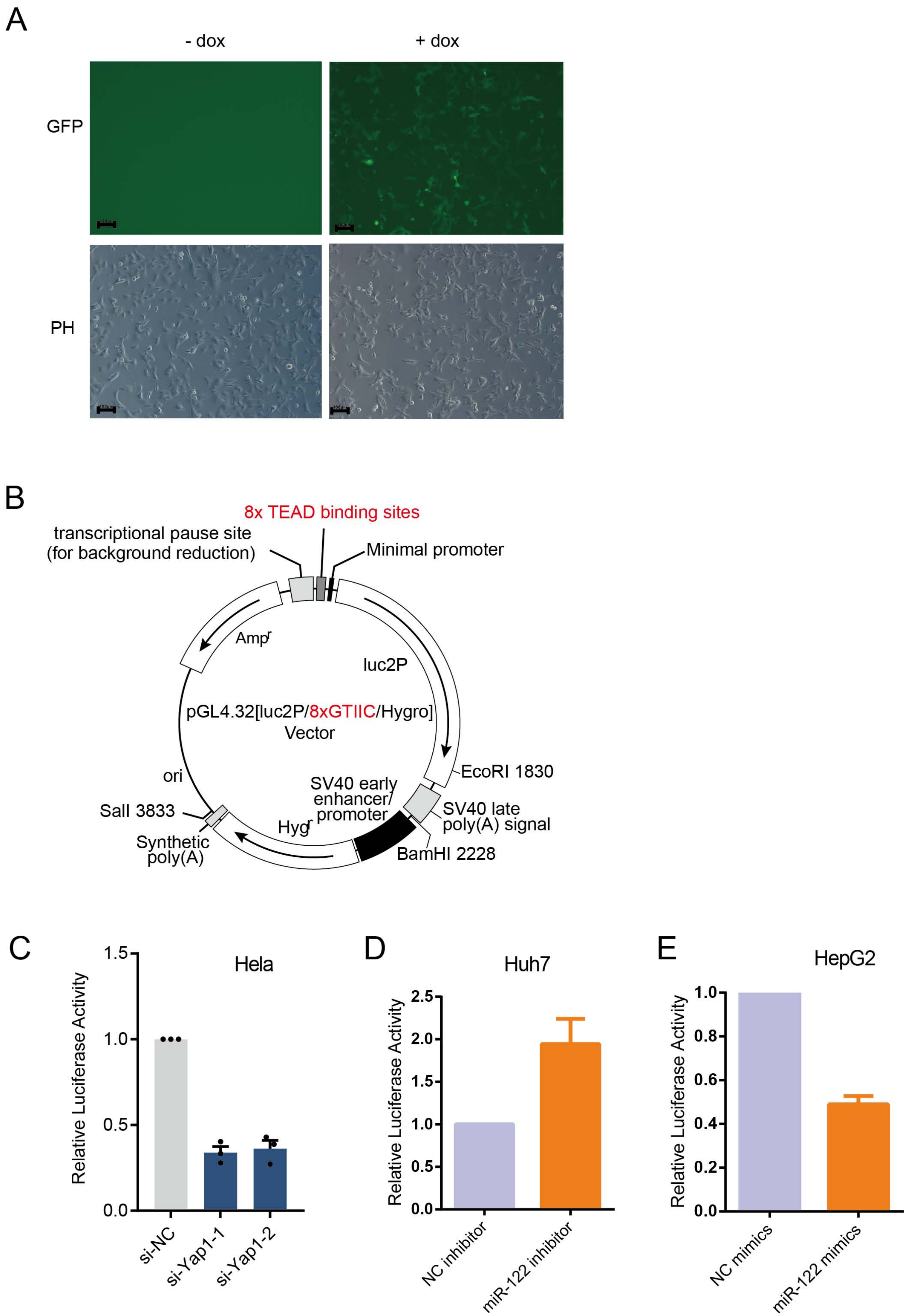
A
- dox
+ dox
GFP
PH
B
8x TEAD binding sites
transcriptional pause site
(for background reduction)
Minimal promoter
Amp^r
luc2P
pGL4.32[luc2P/8xGTIIC/Hygro]
Vector
ori
EcoRI 1830
SV40 early
enhancer/
promoter
SV40 late
poly(A) signal
SalI 3833
Hyg^r
Synthetic
poly(A)
BamHI 2228
C
Hela
Relative Luciferase Activity
1.5
1.0
0.5
0.0
si-NC
si-Yap1-1
si-Yap1-2
D
Huh7
Relative Luciferase Activity
2.5
2.0
1.5
1.0
0.5
0.0
NC inhibitor
miR-122 inhibitor
E
HepG2
Relative Luciferase Activity
1.0
0.8
0.6
0.4
0.2
0.0
NC mimics
miR-122 mimics

Figure S7

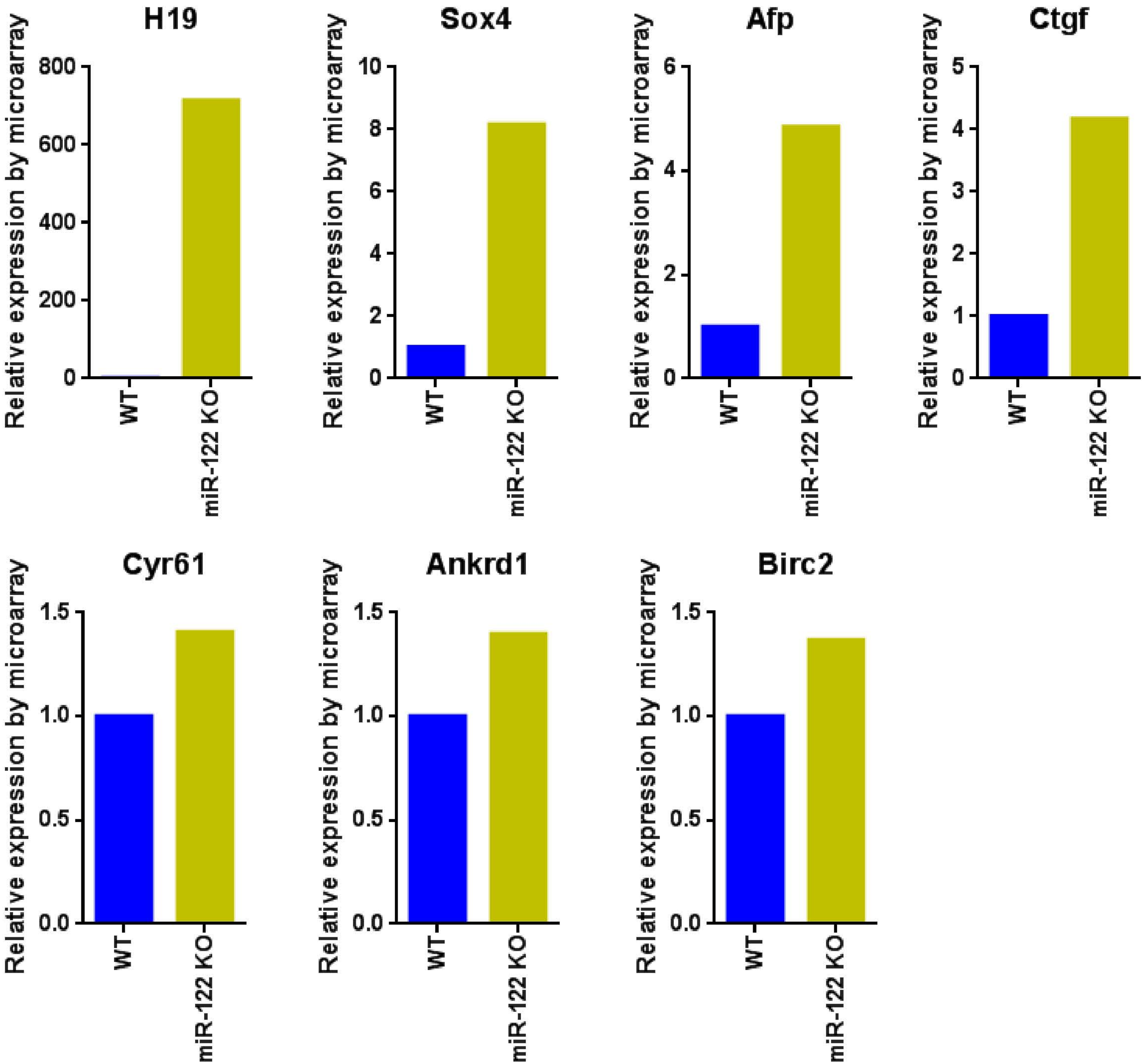
H19
Sox4
Afp
Ctgf
Cyr61
Ankrd1
Birc2
Relative expression by microarray
WT
miR-122 KO
800
600
400
200
0
10
8
6
4
2
5
3
1
1.5
1.0
0.5
0.0

Figure S8

```
Taz-MT:      5'GUCUGCCUUUGUACUCAGUGAGGU 3'
                  : :||  |||    |||
miR-122:     3'UUGUGGUAAC ---AGUGUGAGGU 5'
                    ||  |||    |||||||||
Taz-WT:      5'GUCUGCCUUUGUACUCACACUCCU 3'
```

```
Ppp1cc-MT:   5'CCCAGACUAUCUGAAGCAGUGAGGU 3'
                 |  |  ||:|| ||    ||
miR-122:     3'GUUUGUGGUA-AC--AGUGUGAGGU 5'
                 |  |  ||:|| ||   ||||||||
Ppp1cc-WT:   5'CCCAGACUAUCUGAAGCACACUCCU 3'
```

```
Net1-MT:     5'AGAAAUCCUUAGACAGUGAGGC 3'
                :||   || |     ||
miR-122:     3'GUUUGUGGUAACAGUGUGAGGU 5'
                :||   || |  |  ||||||||
Net1-WT:     5'AGAAAUCCUUAGACACACUCCC 3'
```

```
Arhgap32-MT: 5'GAAACAUGUCUGGACACGUGAGGA 3'
                 |||||:  :  |  |  ||       |
miR-122:     3'GUUUGUG-GUAACAGU-GUGAGGU 5'
                 |||||:  :  |  |  ||  ||||||
Arhgap32-WT: 5'GAAACAUGUCUGGACACCACUCCA 3'
```

```
Ppp2r3a-MT:  5'CUCUCACUAU--ACAGUGAGGU 3'
                 |   |||:||    ||
miR-122:     3'GUUUGUGGUAACAGUGUGAGGU 5'
                 |   |||:||    ||||||||
Ppp2r3a-WT:  5'CUCUCACUAU--ACACACUCCG 3'
```

```
Arhgap5-MT:  5'AGUAUCCUGUUCAUAUGUGAGGU 3'
                  :  ||||:||     ||
miR-122:     3'GUUUGUGGUAA-CAGUGUGAGGU 5'
                  :  |:::|:||    ||||||||
Arhgap5-WT:  5'AGUAUCCUGUUCAUAGCACUCCA 3'
```

```
Rock2-MT:    5'GCACUGGAUGCAAUAGUGAGGU 3'
                  |  ::         :|
miR-122:     3'GUUUGUGGUAACAGUGUGAGGU 5'
                  |  ::         :||||||||
Rock2-WT:    5'GCACUGGAUGCAAUACACUCCA 3'
```

```
Smurf2-MT:   5'AGGACA-CACUUACAGUGAGGU 3'
                 ::|||  ||  |    ||
miR-122:     3'GUUUGUGGUAACAGUGUGAGGU 5'
                 ::|||  ||  |    ||||||||
Smurf2-WT:   5'AGGACA-CACUUACACACUCCU 3'
```

```
Scrib-MT:    5'GUAGGAGGUUCUGAUGUGAGGU 3'
                  |:  |    |    |
miR-122:     3'GUUUGUGGUAACAGUGUGAGGU 5'
                  |:  |    |    | ||||||
Scrib-WT:    5'GUAGGAGGUUCUGAUCACUCCA 3'
```

```
Arhgap11a-MT: 5'AAGGAACCAGCUAAGGUGAGGU 3'
                 |    |||:||    ||
miR-122:      3'GUUUGUGGUAACAGUGUGAGGU 5'
                 |    |||:||    ||||||||
Arhgap11a-WT: 5'AAGGAACCAGCUAAGCACUCCA 3'
```

```
Ppp1r9a-MT:  5'CUGUGACUUUAUUAAGUGAGGU 3'
                 |  :  ||:||    ||
miR-122:     3'GUUUGUGGUAACAGUGUGAGGU 5'
                 |  :  ||:||    ||||||||
Ppp1r9a-WT:  5'CUGUGACUUUAUUAACACUCCA 3'
```

```
Ppp2r4-MT:   5'CUGGCCCCUUUGCGGAAGUGAGGU 3'
                  ::  |  ||  ||
miR-122:     3'GUUUGUGGUAA--CAGUGUGAGGU 5'
                  ::  |  ||  ||   |   |||||||
Ppp2r4-WT:   5'CUGGCCCCUUUGCGGAACACUCCA 3'
```

```
Iqgap1-MT:   5'GGUGCCUCAGU-UCAGUGAGGU 3'
                   :|  :||  |  |||
miR-122:     3'GUUUGUGGUAACAGUGUGAGGU 5'
                   :|  :||  |  |||||||||
Iqgap1-WT:   5'GGUGCCUCAGU-UCACACUCCC 3'
```

```
Foxo1-MT:    5'UGACUACCUUUGUUUGGGGUGAGGU 3'
                  :|  :|||  ||||:    :
miR-122:     3'GUUUGUGGUAACAG---UGUGAGGU 5'
                  :|  :|||  ||||:    :||||||
Foxo1-WT:    5'UGACUACCUUUGUUUGGGCACUCCA 3'
```

```
Ppp2r2a-MT:  5'ACAGUUGGCUGUAAUGUGAGGU 3'
                  |::     |
miR-122:     3'GUUUGUGGUAACAGUGUGAGGU 5'
                  |::     |      ||||||
Ppp2r2a-WT:  5'ACAGUUGGCUGUAAUCACUCCU 3'
```

```
Agt-MT:      5'UGACUACCUUUGUUUGGGGUGAGGU 3'
                  :|  :|||  ||||:    :
miR-122:     3'GUUUGUGGUAACAG---UGUGAGGU 5'
                  :|  :|||  ||||:    :||||||
Agt-WT:      5'agAACCCCAGTGTGGAGACACTCCc 3'
```

```
Rapgef6-MT:  5'ACAGUUGGCUGUAAUGUGAGGU 3'
                  |::     |
miR-122:     3'GUUUGUGGUAACAGUGUGAGGU 5'
                  |::     |      ||||||
Rapgef6-WT:  5'ACAGUUGGCUGUAAUCACUCCU 3'
```

```
Ppp1cb-MT:   5'UCCUGACCUUUGAGUAUCUGGUGAGGU 3'
                    |||  |||     ||  :
miR-122:     3'GUUUGUGGUAAC----AG-UGUGAGGU 5'
                    |||  |||     ||  :||||||
Ppp1cb-WT:   5'UCCUGACCUUUGAGUAUCUGCACUCCC 3'
```

```
Magi3-MT:    5'CAAGAAAGCACCCAUCACUCCA 3'
                  |:  |
miR-122:     3'GUUUGUGGUAACAGUGUGAGGU 5'
                  |:  |         ||||||
Magi3-WT:    5'CAAGAAAGCACCCAUCACUCCA 3'
```

Figure S9

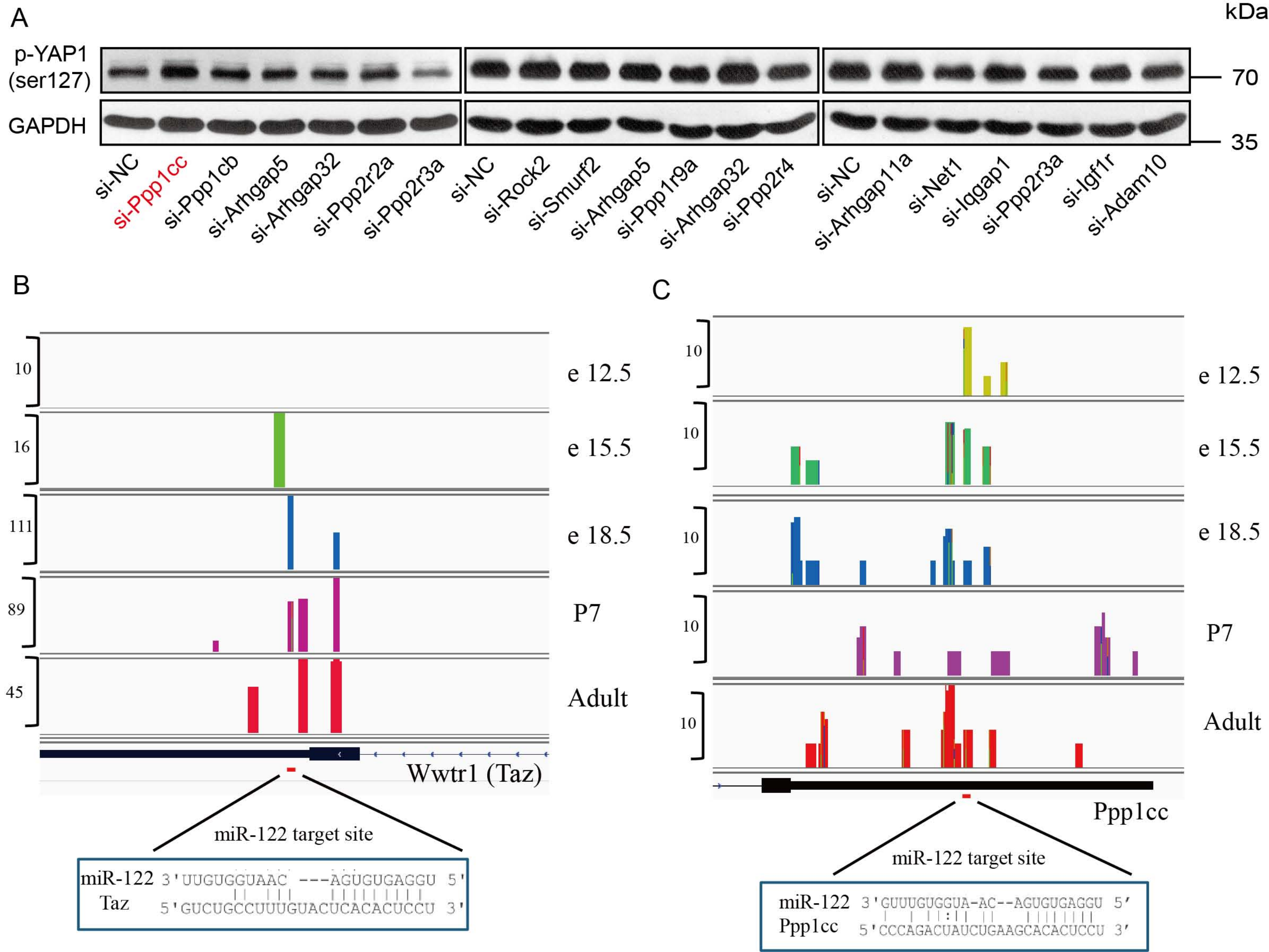
A
kDa
p-YAP1 (ser127)
GAPDH
70
35
si-NC
si-Ppp1cc
si-Ppp1cb
si-Arhgap5
si-Arhgap32
si-Ppp2r2a
si-Ppp2r3a
si-NC
si-Rock2
si-Smurf2
si-Arhgap5
si-Ppp1r9a
si-Arhgap32
si-Ppp2r4
si-NC
si-Arhgap11a
si-Net1
si-Iqgap1
si-Ppp2r3a
si-Igf1r
si-Adam10
B
10
16
111
89
45
e 12.5
e 15.5
e 18.5
P7
Adult
Wwtr1 (Taz)
miR-122 target site
miR-122 3'UUGUGGUAAC ---AGUGUGAGGU 5'
Taz 5'GUCUGCCUUUGUACUCACACUCCU 3'
C
10
10
10
10
10
e 12.5
e 15.5
e 18.5
P7
Adult
Ppp1cc
miR-122 target site
miR-122 3'GUUUGUGGUA-AC--AGUGUGAGGU 5'
Ppp1cc 5'CCCAGACUAUCUGAAGCACACUCCU 3'
