# Supplementary material for: Genome-wide identification of microRNA targets reveals positive regulation of the Hippo pathway by miR-122 during liver development
Source: Cell Death Dis. 2021 Dec 14;12(12):1161. doi: 10.1038/s41419-021-04436-7 (PMC8671590; doi:10.1038/s41419-021-04436-7)
Supplement: Supplementary file 2 — Table S1 [file 41419_2021_4436_MOESM2_ESM.docx]

Table S1 HITS-CLIP reads mapped to mouse genome and transcriptome

| sample | Total reads | Mapping rate (genome) | Mapping rate (transcriptome) |
| --- | --- | --- | --- |
| e12.5 | 67,897,472 | 79% | 74% |
| e15.5 | 26,518,252 | 79% | 75% |
| e18.5 | 22,049,975 | 72% | 68% |
| P7 | 20,229,361 | 80% | 78% |
| Adult | 30,524,418 | 90% | 87% |
| e18.5 rep2 | 94,032,967 | 82% | 77% |
