# Supplementary material for: Genome-wide identification of microRNA targets reveals positive regulation of the Hippo pathway by miR-122 during liver development
Source: Cell Death Dis. 2021 Dec 14;12(12):1161. doi: 10.1038/s41419-021-04436-7 (PMC8671590; doi:10.1038/s41419-021-04436-7)
Supplement: Supplementary file 3 — Table S2 [file 41419_2021_4436_MOESM3_ESM.docx]

Table S2 miRNA-mRNA interaction Relationships of each sample

| sample | miRNA-targets  total | miRNA-targets  in 3’UTR | miRNA-targets  in CDS | miRNA-targets  in 5’UTR |
| --- | --- | --- | --- | --- |
| e12.5 | 431,719 | 161,743 | 245,779 | 24,197 |
| e15.5 | 161,892 | 61,358 | 95,602 | 4,932 |
| e18.5 | 156,427 | 56,686 | 94,053 | 5,688 |
| P7 | 347,106 | 119,466 | 213,261 | 14,379 |
| Adult | 262,941 | 65,965 | 186,390 | 10,586 |
| e18.5 rep2 | 91,404 | 52,300 | 36,263 | 2,841 |
