# Supplementary material for: Genome-wide identification of microRNA targets reveals positive regulation of the Hippo pathway by miR-122 during liver development
Source: Cell Death Dis. 2021 Dec 14;12(12):1161. doi: 10.1038/s41419-021-04436-7 (PMC8671590; doi:10.1038/s41419-021-04436-7)
Supplement: Supplementary file 6 — Table S5 [file 41419_2021_4436_MOESM6_ESM.docx]

Table S5 number and repeatability of miR-122 targets

| Detected samples | 3’UTR | CDS | 5’UTR |
| --- | --- | --- | --- |
| 6 | 25 | 92 | 2 |
| ≥5 | 67 | 189 | 4 |
| ≥4 | 134 | 321 | 13 |
| ≥3 | 253 | 555 | 34 |
| ≥2 | 458 | 963 | 99 |
| ≥1 | 927 | 1,649 | 340 |
