# Supplementary material for: Genome-wide identification of microRNA targets reveals positive regulation of the Hippo pathway by miR-122 during liver development
Source: Cell Death Dis. 2021 Dec 14;12(12):1161. doi: 10.1038/s41419-021-04436-7 (PMC8671590; doi:10.1038/s41419-021-04436-7)
Supplement: Supplementary file 9 — Table S8 [file 41419_2021_4436_MOESM9_ESM.docx]

Table S8-1 Sequence of siRNA and miRNA mimics

| Name | Sense sequences |
| --- | --- |
| Negative control | UUCUCCGAACGUGCAGGUTT |
| miR-122-5p mimics | UGGAGUGUGACAAUGGUGUUUG |
| Mouse si-Yap1 1# | GUUGAAACAACAGGAAUUATT |
| Mouse si-Yap1 2# | GGAGAAGUUUACUACAUAATT |
| Mouse si-Taz 1# | CAGCCGAAUCUCGCAAUGATT |
| Mouse si-Taz 2# | GAUGAAUCCGUCCUCGGUGTT |
| Human si-Yap1 1# | GCUUAUAAGGCAUGAGACATT |
| Human si-Yap1 2# | CCACCAGUGCAGCAGAAUATT |
| Mouse si-PPP1CC-1#  Mouse si-PPP1CC-2# | CGAUGUUGUUUCUGUGGAATT  AUCUUUGGAGCCACUGAUUTT |

Table S8-2 The sequence of primer for qPCR

| Name | Sequences |
| --- | --- |
| U6-F | CGCTTCGGCAGCACATATAC |
| U6-R | TTCACGAATTTGCGTGTCAT |
| RT-miR-122-5p | CTCAACTGGTGTCGTGGAGTCGGCAATTCAGTTGAGCAAACACC |
| FP-miR-122-5p | TCGCCTGGAGTGTGACAATGG |
| Gapdh-F | CGTCCCGTAGACAAAATGGT |
| Gapdh-R | TCAATGAAGGGGTCGTTGAT |
| Taz-F | CAGTCCTATGACGTGACCGA |
| Taz-R | GGTCTTGCCATGTGGTGATT |
| Yap1-F | CCTGATGATGTACCACTGCC |
| Yap1-R | GCCATGTTGTTGTCTGATCG |
| Afp-QF | GGCGATGGGTGTTTAGAAAG |
| Afp-QR | CAGCAGCCTGAGAGTCCATA |
| Ppp1cc-F | CATCGACAGCATCATCCAAC |
| Ppp1cc-R | CGAGACTTCAGGCAGAGTCC |
| Ankrd1-QF | TGAGGCTGAACCGCTATAAGA |
| Ankrd1-QR | CAGTGCAACACCAGATCCAT |
| Cyr61-QF | CACCGCTCTGAAAGGGATCT |
| Cyr61-QR | TTTACAGTTGGGCTGGAAGC |

Table S8-3 Adaptor and primer used for HITS-CLIP(N is barcode sequence)

| Name | sequences |
| --- | --- |
| 3’ RNA linker | AMP-5’p=5’pCTGTAGGCACCATCAATdideoxyC-3’ |
| 5’ RNA linker | 5’GUUCAGAGUUCUACAGUCCGACGAUCNNNNAC’3 |
| RT primer | 5’-ATTGATGGTGCCTACAG-3’ |
| Sol_3_Modban | 5'-CAAGCAGAAGACGGCATACGATTGATGGTGCCTACAG-3' |
| Sol_5_SBS3 | 5'-AATGATACGGCGACCACCGACAGGTTCAGAGTTCTACAGTCCGA-3' |
